# Transcriptional landscape and essential genes of *Neisseria gonorrhoeae*

**DOI:** 10.1093/nar/gku762

**Published:** 2014-08-20

**Authors:** Christian W. Remmele, Yibo Xian, Marco Albrecht, Michaela Faulstich, Martin Fraunholz, Elisabeth Heinrichs, Marcus T. Dittrich, Tobias Müller, Richard Reinhardt, Thomas Rudel

**Affiliations:** 1 Department of Bioinformatics, University of Würzburg, 97074 Würzburg, Germany; 2 Department of Microbiology, University of Würzburg, 97074 Würzburg, Germany; 3 Max Planck-Genome-centre Cologne at MPI for Plant Breeding Research, 50829 Cologne, Germany

## Abstract

The WHO has recently classified *Neisseria gonorrhoeae* as a super-bacterium due to the rapid spread of antibiotic resistant derivatives and an overall dramatic increase in infection incidences. Genome sequencing has identified potential genes, however, little is known about the transcriptional organization and the presence of non-coding RNAs in gonococci. We performed RNA sequencing to define the transcriptome and the transcriptional start sites of all gonococcal genes and operons. Numerous new transcripts including 253 potentially non-coding RNAs transcribed from intergenic regions or antisense to coding genes were identified. Strikingly, strong antisense transcription was detected for the phase-variable *opa* genes coding for a family of adhesins and invasins in pathogenic *Neisseria*, that may have regulatory functions. Based on the defined transcriptional start sites, promoter motifs were identified. We further generated and sequenced a high density Tn5 transposon library to predict a core of 827 gonococcal essential genes, 133 of which have no known function. Our combined RNA-Seq and Tn-Seq approach establishes a detailed map of gonococcal genes and defines the first core set of essential gonococcal genes.

## INTRODUCTION

*Neisseria gonorrhoeae* (GC) is the cause of gonorrhoea, one of the most frequent sexually transmitted diseases. The obligate human-specific pathogens colonize the mucosal surface of the urogenital tract but may also disseminate into the blood stream to cause disseminated gonococcal disease. The incidence of gonococcal infections increased in the last years to more than 100 million annually (1). In addition, multi-resistant gonococci spread globally which may result in non-treatable gonorrhoea in the near future.

Many different factors have been characterized in gonococci that support the pathogenic potential of GC. Type IV pili mediate the initial attachment to mucosal cells and are therefore important colonization factors (2). Proteins in the outer membrane like the family of opacity-associated (Opa) proteins mediate the initial attachment to, invasion into and traversal through epithelial cells (3,4). To acquire iron, GC takes up host transferrin and lactoferrin by membrane-located transferrin and lactoferrin binding proteins. GC are naturally competent for the uptake of their own DNA which is recognized by virtue of the small 10 bp 5′-GCCGTCTGAA DNA uptake sequence (DUS) (5). Approximately 2000 copies of the DUS are present in GC, indicating a frequent uptake and integration of free DNA into the genome (6,7).

The genome of GC consists of about 2.24 Mb and contains 2185 annotated genes (Broad Institute, release date 10/19/2012). Similar as in other naturally competent bacteria, the gonococcal genome is highly dynamic and genomic inversions, deletions and substitutions have been frequently observed. Although extensive transcriptional regulation accompanies the infection process of GC, only 34 putative transcriptional regulators (compared to more than 200 in *Escherichia* *coli*) are found in *N. gonorrhoeae* (strain FA1090 GenBank AE004969) (8). This points at a limited transcriptional regulation within the obligate human pathogen, potentially caused by stable growth conditions within their restricted niches. Of the 34 potential transcriptional regulators, only 9 have been described in detail. Fur is the major regulator for the adaptation to iron-stress conditions (9), fumarate and nitrate reduction regulator (FNR) functions as an oxygen-sensing transcription factor (10), whereas NsrR is induced by nitric oxide and is important for the regulation of genes responsible for nitric oxide metabolism (11). RegF was identified to negatively regulate expression of gonococcal *pilE*, coding for the major subunit of the type IV pili (12). In addition, a MerR-like transcription factor, termed NmlR (*Neisseria* MerR-like Regulator), was shown to be an important regulator for survival of GC in the presence of reactive nitrogen species (13). GC possess two regulators specialized for peroxide stress response, the LysR-like transcriptional regulator OxyR, which negatively controls expression of catalase (14,15), and PerR, a metalloregulatory protein-like Fur (16), which controls the manganese-dependent stress response to reactive oxygen species (17). Finally, gonococci harbor two transcriptional regulators responsible for the control of efflux pump systems for hydrophobic compounds: the multiple transferrable resistance regulator MtrR controls expression of the MtrCDE efflux pump for hydrophobic agents (18) and additionally influences the gonococcal susceptibility to penicillin (19), whereas the fatty acid resistance regulator FarR represses transcription of the FarAB efflux pump providing resistance against long-chain fatty acids (20).

Similar as with transcriptional regulators, the diversity of sigma factors present in GC is limited. RpoD (σ70) represents the GC house-keeping sigma factor but several alternative sigma factors have been described. RpoH (σ32), an alternative sigma factor for heat shock response in GC and the initial steps of an infection has been shown to be essential for GC (21). The alternate sigma factor RpoN (22–24) may not be functional as its deduced sequence lacks the Helix–Turn–Helix motif necessary for DNA binding (25–27). The *ecf* gene encodes a member of the extracytoplasmic function (ECF) family of sigma factors which respond specifically to a variety of extracytoplasmic stimuli. Ecf in gonococci controls a small regulon which contains the *msr*AB gene that encodes an unusual methionine sulfoxide reductase (28).

Very few small regulatory RNAs (sRNAs) have been identified in *Neisseria*. A Fur-regulated sRNA, called NrrF, has been identified in meningococci (29) and gonococci (30), that was upregulated under iron depleted conditions. NrrF has been shown to be involved in the regulation of the *sdh*A and *sdh*C genes (subunits of the succinate dehydrogenase complex). Further transcriptional analysis revealed that NrrF regulates the *sdh* operon in an Hfq-dependent manner (31). A σE-dependent sRNA of unknown function has been identified in *N. meningitidis* (32). Another sRNA, AniS in meningococci (and the gonococcal homologue FnrS) is synthesized under oxygen limiting conditions (33,34). Recently, a sRNA was identified in GC that acts in *cis* and influences antigenic variation of *pilE* (35).

Here, we used differential RNA sequencing (dRNA-Seq) and transposon insertion site (TIS) sequencing (Tn-Seq) to map the GC transcriptome and identify genes essential for gonococcal survival. We mapped transcriptional start sites (TSS) of annotated genes, identified novel transcripts comprising candidate non-coding RNAs, polycistronic transcripts as well as promoter sequences. By massively parallel sequencing of a highly complex Tn5 transposon mutant library we identified numerous essential genes, which may provide new targets for anti-gonococcal therapy.

## MATERIALS AND METHODS

### Bacterial strains and growth conditions

*N. gonorrhoeae* strain MS11 (GenBank accession number NC_022240.1) and the derivative strain N2009 ((36), P^+^, Opa^−^, PorB_IA_, Cam^r^, Erm^r^) were grown on GC agar plates (Oxoid), supplemented with vitamine mix (1%) and, if applicable, kanamycin (40 mg/l) for 14–16 h at 37°C in a humidified 5% CO_2_ atmosphere. For transformation of *N. gonorrhoeae*, piliated bacteria were selected by colony morphology (37) under a stereo microscope. 5 × 10^6^ bacteria were mixed with 10 ng polymerase chain reaction (PCR) products in 50 μl PPM medium (15 g Proteose peptone; 5 g sodium chloride; 0.5 g soluble starch; 1 g potassium dihydrogen phosphate; 4 g dipotassium hydrogen phosphate for 1 l; pH 7.2; 1% vitamin mix, 0.5% sodium hydrogen carbonate, 10 mM magnesium chloride, sterilized by filtration), and spotted onto a GC agar plate. After overnight incubation, the bacteria were resuspended in PPM medium and plated onto GC agar plates containing kanamycin for selection of transformants. For RNA extraction, GC were grown in PPM medium as described for transformation at an OD of 0.2 and grown at 180 revolutions per minute (rpm) at 37°C to an OD 0.4–0.5 (mid-logarithmic phase).

### Isolation of bacterial RNA

Bacteria either grown on GC agar plates or in PPM medium to the mid-logarithmic phase were resuspended in phosphate buffered saline (PBS) or harvested by centrifugation, respectively. Bacterial pellets were washed twice in ice cold PBS, resuspended in 1 ml of TRIzol reagent (Life Technologies) and frozen at −80°C. RNA isolation was performed according to TRIzol RNA isolation procedure after mechanical disruption of bacteria using a FastPrep-24 System and Lysing Matrix B tubes (MP Biomedicals). Briefly, after addition of 0.2 ml chloroform, vigorous shaking and incubation at room temperature for 2 min, the samples were centrifuged (12 000 *g*, 15 min, 4°C). The aqueous phase was mixed with 0.5 ml isopropanol for RNA precipitation. After centrifugation the pellet was washed with 1 ml 75% ethanol, dried and then dissolved in RNase-free water. Quality of the bacterial RNA was measured using a Bioanalyzer 2100 (Agilent).

### Preparation of cDNA libraries for dRNA-Seq

dRNA-Seq analyses were performed in *N. gonorrhoeae* strain MS11 by pyrosequencing (454, Roche) and Illumina sequencing. cDNA cloning for pyrosequencing was done as described before (38) but omitting size fractionation of RNA prior to cDNA synthesis. Equal amounts of total RNA were used for the generation of all cDNA libraries. Primary transcripts of total RNA were enriched by selective degradation of RNAs containing 5′ mono-phosphate (5′P) by treatment with a 5′ P-dependent Terminator exonuclease (TEX; Epicentre Biotechnology) (39). Primary bacterial transcripts (most mRNAs and sRNAs) are protected from exonucleolytic degradation by their tri-phosphate (5′PPP) RNA ends. For linker ligation RNA was treated with tobacco acid pyrophosphatase to generate 5′-mono-phosphates. After addition of specific 5′-linkers with unique tags for each library and poly-A-tailing, the RNA was converted into a cDNA library. cDNA libraries were generated from total RNA and total RNA enriched for primary transcripts from bacteria grown in PPM medium from two biological replicates. These four libraries were then sequenced on a Roche/454 GS-FLX system using the ‘Amplicon A’ and the ‘SR70 sequencing kits’.

A second set of cDNA libraries was prepared from GC grown on GC agar plates and subjected to high throughput sequencing on the Illumina platform. Bacterial colonies from different incubation times (12–48 h) were collected and resuspended in ice cold PBS. An equivalent of 50 OD_550_ bacteria (∼1.5 × 10^10^) was collected by centrifugation (4000 rpm, 4°C) and resuspended in 0.8 ml of lysis buffer (20 mM Tris pH 8, 150 mM KCl, 1 mM MgCl_2_, 1 mM DTT), and disrupted with 0.8 ml glass beads (Roth, 0.1 mm diameter) by rigorous vortexing (30 s burst followed by 30 s chill on ice) for 5 min. The cleared lysate was subjected to DNase I digestion using 1U DNase per μg, a portion of RNA in the supplied reaction buffer for 30 min at 37°C. RNA was then isolated by phenol/chloroform/isoamyl alcohol (PCI) extraction and precipitation of RNA by 2.5 volumes of ethanol containing 0.1 M sodium acetate at −20°C for 60 min and a washing step with 80% ethanol at −20°C. RNA was treated with DNase I to digest remaining DNA. Therefore, RNA was incubated with 1U DNase I (Fermentas) per μg of RNA in the supplied reaction buffer for 30 min at 37°C and subsequently extracted by another PCI extraction step. Half of the amount of RNA was treated with TEX (Epicentre Biotechnology) according to the manufacturer's protocol followed by another PCI extraction. Both, TEX-treated and TEX-untreated RNA were then used to construct cDNA libraries by Vertis Biotechnologie AG (Munich, Germany), which were subsequently sequenced on a Solexa GAIIx machine.

### Analysis of dRNA-Seq data

Adapter, primer and poly A sequences were trimmed from reads of all dRNA-Seq libraries via cutadapt 1.2.1 (40) if there was a minimum overlap of five nucleotides at read ends with a maximum mismatch ratio of 0.1 between sequence patterns and reads. The resulting reads with less than 14 remaining bases were excluded from further analyses. The removed sequence pattern for Illumina dRNA-Seq libraries was ‘AAAAAAAAAAAA’. Additionally, the adapter sequence ‘GACCTTGGCTGTCACTCA’ was removed in Roche 454 dRNA-Seq libraries. Curated sequence reads were mapped on the ring chromosome (2 233 640 bp) and the plasmid (4153 bp) sequences of *N. gonorrhoeae* MS11 version 4 (*N. gonorrhoeae* group Sequencing Project, Broad Institute of Harvard and MIT (http://www.broadinstitute.org/)) via Bowtie 2.0.2 (41). Mapping parameters were set for ‘very-sensitive global’ alignments. Suboptimal alignments and alignments with three or more mutations between reads and reference genome were removed to reduce the false read mapping rate.

Curated read alignments were used to calculate read coverage along the reference genome representing the number of reads covering a base using the R package ‘IRanges’ version 1.18.3. For reads mapping to multiple positions, each location was proportionally weighted according to the number of mapping positions. Graph files containing the coverage tracks for each dRNA-Seq library were generated for the plus and minus strand. The computed coverage tracks of the eight dRNA-Seq libraries (2× TEX-treated 454, 2× untreated 454, 2× TEX-treated Illumina, 2× untreated Illumina) were visualized in parallel along the genome using IGB 7.0.3 (42). TSS and transcriptional end sites (TES) were identified manually by examining characteristic expression profiles in at least two tracks for each transcript. Information of manually annotated TSS and TES were combined with coding sequence (CDS) annotated by the Broad institute. For further analyses, the gene starts were defined either by TSS (if a TSS was identified and the gene is the first gene in the transcript) or alternatively by the CDS start. The gene ends were defined by the CDS end (if a CDS annotation was available) or alternatively by the TES. For the Illumina dRNA-Seq library, gene expression was quantified and normalized as reads per kilobase of gene model per million of mapped reads (RPKM) (43). The RPKM cutoff to classify a genomic region of interest as expressed was defined as 75% quantile of RPKM values of intergenic regions.

### Riboswitch detection

To detect riboswitches in the MS11 genome, covariance models containing RNA sequence and structure models of the Rfam 11.0 database (44) were downloaded, including 26 different riboswitch families. Cmscan of Infernal 1.1 package (45) was used to search the MS11 genome, using the default scoring. Hits with an e-value less than 1.0 were reported.

### Analysis of promoters

For the promoter analyses sequences of the promoter regions (−1 to −70 upstream of annotated TSS) have been extracted from genes with annotated TSS. Only promoter regions of protein coding genes (comprising a CDS) with manually annotated primary transcription start sites have been included, yielding a set of 890 promoters of length 70 bp. This sequence set has been used for all subsequent analyses. To identify significantly enriched sequence patterns (motifs) in the promoter regions, we used MEME (Multiple EM for Motif Elicitation; (46)). This algorithm discovers *de novo* significantly overrepresented conserved regions. Briefly, motifs are represented by gapless position-dependent base-probability matrices which describe the probability of each possible base at each position in the pattern. In contrast to motif search approaches where the position of *a priori* given motifs are searched in the sequences, here using a motif discovery algorithm the position of the motif and the motif itself (position-specific base frequencies) are identified simultaneously. For the motif analysis a local installation of MEME version 4.9.1 (46,47) was established. A zero or one occurrence model (zoops, assuming that each distinct motif occurs maximally one time in a promoter) was chosen and motifs between 5 and 20 bases in length have been searched using an E-value threshold of 0.001 where a maximal number of 10 motifs was allowed.

In the similarity network analysis all promoter sequences were compared by all against all pairwise optimal local alignments using the Smith–Waterman algorithm as implemented in Biostrings using R (http://www.R-project.org/, version 3.0.1). Prior to the alignment calculations of the DUS ‘TTCAGACGGCAT’ and its reverse complement have been masked in all promoter sequences. Due to the compositional bias in the promoter sequences (overrepresentation of A/T versus G/C, Supplementary Figure S3B) a composition adjusted scoring matrix based on the Felsenstein model (48) has been estimated (see Supplementary Figure S3B). For all alignments linear gap costs of −7 were used. For all alignment scores empirical *P*-values were calculated based on background scores derived from pairwise alignments of randomly sampled sequences with the same base composition and length (Supplementary Figure S3C). All observed scores above a threshold the 99.999% quantile of the background distribution (corresponding to an alignment score of 71.04) have been considered as significant. Multiple sequence alignments of promoter sequences have been calculated using ClustalX (49). Promoter region alignments have been visualized by ESPript (50) and the WebLogo server (51).

### Northern blot analysis of sRNAs

To confirm the expression and size of putative transcripts total RNA from GC was separated on a denaturing 15% polyacrylamide gel containing 8 M urea. Electrophoresis was performed in 1× TBE buffer at 150 V for ∼3 h. After transferring the RNA onto a nylon membrane by wet blotting in 0.5× TBE at 25 V for 1 h, it was covalently cross-linked by ultraviolet irradiation. For the specific detection of putative sRNAs 24-mer DNA oligonucleotides antisense to the RNAs were end labeled with (γ^32^P)-adenosine triphosphate (ATP). Membranes were hybridized with the respective probes (Supplementary Table S5) at 42°C for 6 h in hybridization buffer (Rapid-Hyb, GE Healthcare) and washed twice with washing buffer (2× SSC, 0.1% sodium dodecyl sulphate (SDS)) at 45°C. Blots were exposed to phosphor storage screens (Fujifilm) which were then scanned by Typhoon 9200 imager (GE Healthcare).

### Homology search in *N. gonorrhoeae* FA1090 and NCCP11945

Homology searches were performed in the *N. gonorrhoeae* strains FA1090 and NCCP11945 for the gene annotations based on the combined information of manual TSS and TES, as well as CDS annotations. This was realized via strand-specific local alignments between nucleotide sequence of the genes versus peptide sequences of all known and hypothetical FA1090 and NCCP11945 proteins using NCBI-BLAST 2.2.28+ (52). For each gene, the best hit in the protein database was reported if the E-value was less than 0.001.

### Transposon library construction

*N. gonorrhoeae* N2009 (MS11, P^+^, Opa^−^, PorB_IA_, Cam^r^, Erm^r^) was used for transposon library generation, since this strain was fully transformation competent and in addition carried the PorB_IA_ derivative mediating PorB_IA_-dependent invasion (36). Genomic DNA was extracted using the NucleoSpin Tissue kit (Macherey-Nagel) and 0.5 μg genomic DNA was mutagenized with 0.12 pmol Tn5 transposon *in vitro* (EZ-Tn5^TM^ <KAN-2> Insertion Kit, Epicentre Biotechnologies) and then purified by phenol extraction and ethanol precipitation. The mutagenized DNA was mixed with 1 U T4 DNA polymerase (Fermentas), 2 nmol dNTPs and incubated at 11°C for 20 min followed by heat inactivation at 75°C for 10 min. After phenol extraction and ethanol precipitation, the mutagenized DNA was ligated by 5 U T4 DNA ligase (Fermentas) at 16°C overnight and finally precipitated for transformation. 0.1 μg mutagenized DNA was mixed with 50 μl suspension of *N. gonorrhoeae* N2009 (OD_550nm_ = 0.32) and incubated for 24 h on a GC agar plate and then colonies were transferred to GC agar plates supplemented with kanamycin and incubated for 48 h at 37°C. This was repeated six times resulting in a library harboring about 100 000 individual colonies. The colonies were harvested in PPM medium, mixed with 100% glycerol to a final concentration of 25% (v/v) and then stored at −80°C.

### Tn-Seq sample preparation and Illumina sequencing

The pellet of the mutant library generated in strain N2009 was divided into two parts to generate two separate libraries (library A and library B). The sequencing samples were prepared in parallel from these two libraries to test the reproducibility of the method. In order to isolate neisserial genomic DNA, the neisserial pellet was resuspended in 500 μl GTE buffer containing 200 μg/ml RNase A and 0.1% SDS and was incubated at 42°C for 10 min until the solution was clear. The lysate was transferred to a Phase Lock Gel™ tube (5 PRIME GmbH) and purified by phenol-chloroform extraction and ethanol precipitation. Purified genomic DNA was sheared by sonication (Bandelin Sonorex RK 255S) with 10 pulses of 60 s duration followed by pauses of 30 s. Sheared DNA was blunted and A-tailed by New England Biolabs (NEB) end repair and dA-tailing modules according to the manufacturer's instructions. Equimolar quantities of primers ‘Adaptor sense’ and ‘Adaptor antisense’ (Supplementary Table S5) were mixed in ‘oligo annealing buffer’ (10 mM Tris-HCl, pH 7.5, 100 mM NaCl, 1 mM EDTA). The mixture was heated for 5 min at 94°C and was allowed to slowly cool to room temperature over an hour to form DNA adaptors. Note that 0.4 nmol annealed adaptors were ligated to 0.5 μg A-tailed DNA with T4 DNA ligase (Fermentas) at 16°C overnight. The ligation products in a range of 250–400 bp were size-selected by gel extraction (Fermentas).

Enrichment of DNA fragments containing parts of the transposon was performed by PCR with primers complementary to the adaptor and to the transposon mosaic end sequence (-TnSeq-PE-Index2 for library A, TnSeq-PE-Index3 for library B and P5-ME; Supplementary Table S5) according to the manufacturer's instructions. Both primers contain Illumina-specific sequences that are complementary to two specific capture oligonucleotides on the Illumina flow cell. PCR products of 250–300 bp were size-selected and gel purified prior to Illumina sequencing.

DNA fragments containing transposon mosaic ends were bound on an Illumina flow cell and amplified on the Illumina cluster station. DNA was sequenced on an Illumina HiSeq 2000 sequencer using 101 bp sequence cycles with a sequence primer that binds to the transposon mosaic end (Tn-Seq Primer; Supplementary Table S5). The resulting 101 bp long reads start exactly with the nucleotide next to the TIS. The library-specific barcode is sequenced by Tn-Seq index SP which binds to the adaptor sequence next to barcode (Tn-Seq index SP; Supplementary Table S5).

### Analysis of Tn-Seq data

Adapter, primer and poly A sequences were trimmed from reads of all Tn-Seq libraries via cutadapt 1.2.1 (40) if there was a minimum overlap of five nucleotides at read ends with a maximum mismatch ratio of 0.1 between sequence patterns and reads. The resulting reads with less than 14 remaining bases were excluded from further analyses. Reads of Tn-Seq libraries were trimmed by the Illumina adapter sequence ‘AGATCGGAAGAGCGGTTCAGCAGGAA’. Curated sequence reads of Tn-Seq libraries were mapped simultaneously on the ring chromosome (2 233 640 bp) and the plasmid (4153 bp) sequences of *N. gonorrhoeae* MS11 version 4 (*N. gonorrhoeae* group Sequencing Project, Broad Institute of Harvard and MIT (http://www.broadinstitute.org/)) via Bowtie 2.0.2 (41). Mapping parameters were set for ‘very-sensitive global’ alignments. Suboptimal alignments and alignments with three or more mutations between reads and reference genome were removed to reduce the false read mapping rate.

To identify TIS, alignment start positions of mapped reads were extracted for both Tn-Seq libraries. Each position on the genome, covered by at least one alignment start position, was annotated as TIS. The essentiality of genes was predicted by testing for TIS underrepresentation, assuming as null-hypothesis, that TIS were uniformly distributed over the genome. The identified TIS on the ring chromosome were permuted and for each permutation the number of TIS was counted gene-wise. The permutation and counting steps were repeated 10 000 times. Subsequently, empirical *P*-values were calculated for each gene based on the null distribution. According to Benjamini and Hochberg (53), the *P*-values were corrected for multiple testing, controlling the false discovery rate.

### Site-directed mutagenesis of GC genes and promoters

To delete genes or replace native promoters with Ptrc, *N. gonorrhoeae* strain MS11 was transformed with linear DNA constructs, which recombined into the chromosome and replaced the respective genes with a kanamycin resistance cassette. The DNA constructs were generated in a two-step overlap PCR reaction. First, an ∼500 bp sequence upstream of the transcription start site of the respective sRNA gene was fused to a kanamycin resistance cassette (primer pairs up_s, adding a DUS, and up_as, or Kan_s and Kan_as). In the second step, a 500 bp downstream flanking region of the sRNA gene was added (primers down_s and down_as). Transformants were validated by sequencing and northern blot detection.

### Functional enrichment in essential genes

Protein sequences of (respectively not essential) CDS defined as essential, were mapped on the COG (cluster of orthologous genes) database (prokaryotic proteins) via RPSBLAST to identify COG families and classes (54). COG classes with unknown function or less than five hits were removed from further analyses. To test for enrichment of essential genes in COG classes, Fisher's exact test was performed (55). Resulting *P*-values were corrected for multiple testing (53).

### Genetic footprinting

Genetic footprinting on GC transposed DNA fragments from *in vitro* and *in vivo* libraries was performed to validate a subset of the candidate identified essential genes as described before (56–59). First, the predicted essential gene regions were amplified from *N. gonorrhoeae* MS11 strain N2009 chromosome by PCR reaction. Then, the purified PCR products were transposed *in vitro* by Tn5 transposon and the gaps in transposed products were repaired with T4 DNA polymerase followed by T4 DNA ligase as described above. A part of transposed DNA as *in vitro* library was used for PCR-based footprinting. Then, transposed DNA was transformed into *N. gonorrhoeae* N2009 and the mutants were selected on GC agar plates supplemented with kanamycin. The mutants were collected in PPM medium supplemented with 2.5 mM MgCl_2_ and 0.1 mM CaCl_2_, and incubated with 1 U/ml Dnase I (Fermentas) at 37°C for 30 min to remove remaining extracellular transposed DNA. Subsequently, the mutants were passaged to a new GC-kan plate. After several passages on these selective plates, genomic DNA of the mutants’ pool was isolated by phenol-chloroform extraction and ethanol precipitation as described above. The genomic DNA from the mutants as *in vivo* library was used for PCR-based footprinting. Genetic footprinting was carried out as described (56–59) by using a transposon-specific primer (Tn ME sequence, Supplementary Table S5) and primers specific to each chromosomal region (Supplementary Table S5). PCR reactions consisted of 200 ng transposed DNA fragments from *in vitro* or *in vivo*, 50 pmol of each primer, 10 nmol dNTPs, 2.5 U Taq DNA polymerase (Genaxxon) and 0.4 U Phusion DNA polymerase in 1× buffer S in a 50 μl reaction. The PCR program was as follows: 30 s at 95°C; 30 cycles of 94°C for 30 s, 58°C for 30 s and 68°C for 30 s + 10 s per cycle. PCR products were analyzed by gel electrophoresis on 1% agarose gel.

### Conditional knockout analysis

To validate a subset of putative essential genes conditional knockout assays were performed. For this purpose we exchanged the promoter of the respective genes with an Isopropyl-β-D-thiogalactopyranosid (IPTG)-inducible Ptrc promoter flanked by a kanamycin resistance cassette. The Ptrc promoter was PCR-amplified with the primer pair Ptrc-F and Ptrc-R (Supplementary Table S5) and as a template the Hermes-10 vector (60) was used. The PCR product was subsequently cloned into pGEM-T (Promega) according to the manufacturer's instructions. The kanamycin resistance cassette containing the neisserial DUS (5′-atgccgtctgaa-3′(5,61)) was amplified from pCR2.1-Tn5-DUS using oligonucleotides Kan-SpeI-F and Kan-SacI-R. The PCR fragment was restricted with the endonucleases SpeI and SacI and was inserted in an accordingly restricted pGEM-T-Ptrc resulting in pGEM-T-kan-Ptrc. The kan-Ptrc cassette was amplified with primers Ptrc-R and Kan-cassette-R (Supplementary Table S5) using pGEM-T-kan-Ptrc as a template. Approximately 500 bp long regions upstream and downstream of the targeted promoter were combined with the kan-Ptrc cassette via fusion PCR (rib-up-f and rib-up-r for amplification of upstream region, rib-down-f and rib-down-r for downstream region; Supplementary Table S5). The resulting PCR fragment was purified and used for transformation of *N. gonorrhoeae* MS11 wild-type strain. Bacteria were plated on selective GC plates containing 40 μg/ml kanamycin. Successful promoter replacement was verified by amplifying the respective genomic region via PCR and subsequent sequencing. Afterwards, the mutants were conjugated with *N. gonorrhoeae* MS11 N220 (containing a lacI^q^ expression cassette on pTH10a (60)) and selected on GC agar plates containing 40 μg/ml kanamycin, 7 μg/ml erythromycin and 0.5 mM IPTG. Essentiality of the gene of interest was checked by growth defect when IPTG was omitted.

## RESULTS

### The transcriptome of *N. gonorrhoeae* MS11

The preliminary annotation of the *N. gonorrhoeae* MS11 genome (version 4) consists of 2185 CDS (2176 CDS on the chromosome, 9 CDS on the plasmid) (*N. gonorrhoeae* group Sequencing Project, Broad Institute of Harvard and MIT; http://www.broadinstitute.org/) and 67 automatically annotated ncRNAs. We augmented the MS11 genome annotation and performed a homology search in existing protein coding genes of strains FA1090 (GenBank) or NCCP11945 (GenBank), yielding 2036 and 2232 homologs, respectively. For 304 of the 2576 annotated genomic features, no homologous region was found in either FA1090 or NCCP11945. Interestingly, we identified homologies to protein coding genes in FA1090 or NCCP11945 strains for 26 novel transcripts that were absent in the primary MS11 CDS annotation (Supplementary Table S1).

To define the transcriptome of *N. gonorrhoeae*, RNA was isolated from strain MS11 either grown on GC agar plates or in liquid media for subsequent deep sequencing of total RNA. Treatment of the RNA with TEX enabled the exact determination of TSS at single nucleotide resolution as previously demonstrated for other human-pathogenic bacteria (39).

**Figure 1.**
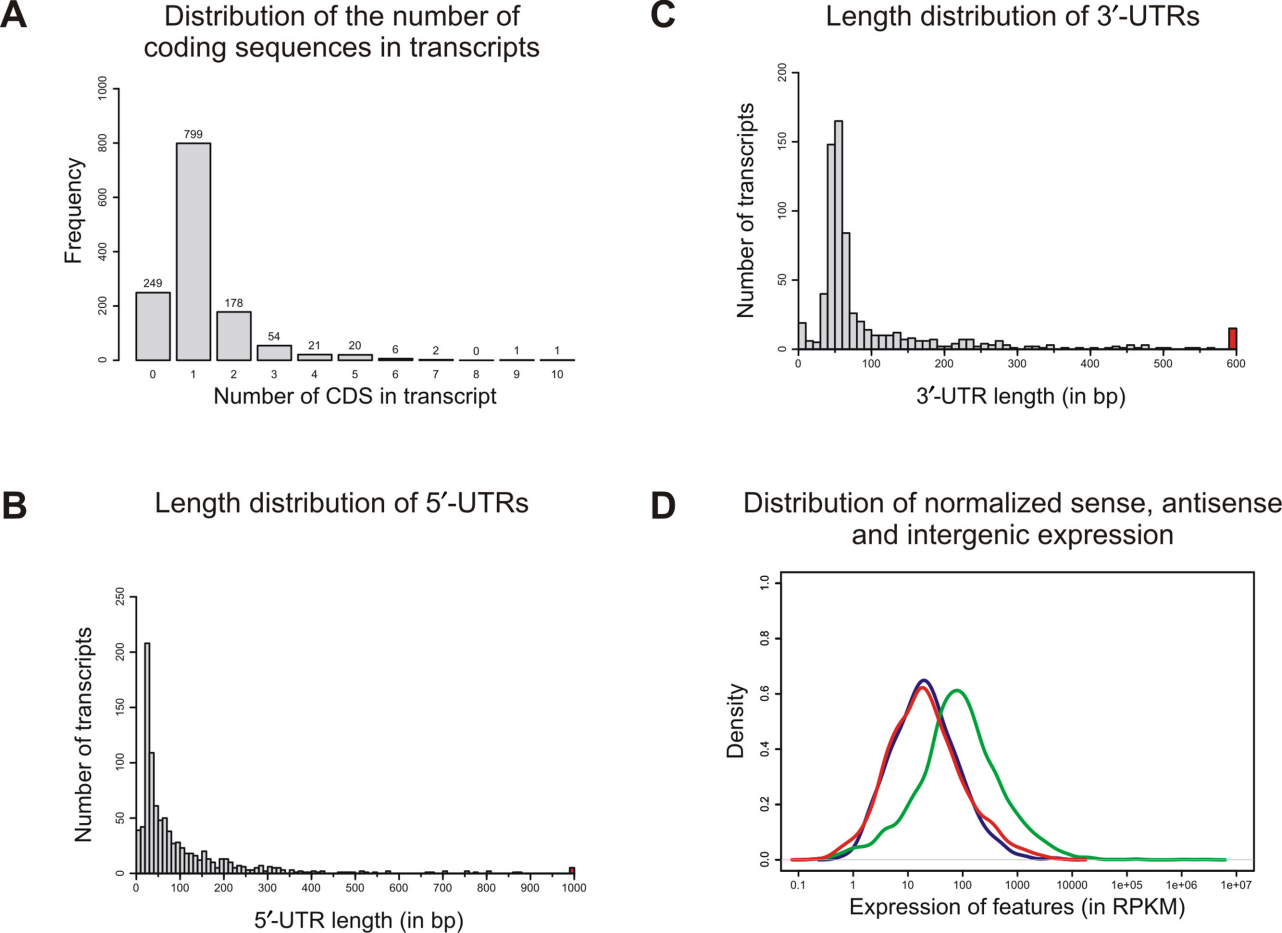
Transcriptome annotation and expression. **(A)** Frequency for transcripts containing different numbers of genes. **(B)** 5′-UTR length distribution of 918 annotated protein coding genes on the ring chromosome for which a distinct primary TSS could be assigned. 5′-UTRs longer than 1000 nt were collapsed into one colored bin. **(C)** 3′-UTR length distribution of 709 annotated protein coding genes for which a distinct TES could be assigned. 3′-UTRs longer than 600 nucleotides were collapsed into one colored bin. **(D)** Expression values in RPKM for genes (green), antisense transcription (red) and intergenic regions (blue).

To achieve a more comprehensive view of the transcription activity and to avoid potential technological restrictions, cDNA libraries were generated from two biological replicates with and without Tex-treatment and analyzed by Illumina and 454 sequencing. Thus, a total of eight cDNA libraries were sequenced by Illumina and 454 technologies resulting in a total of over 17.6 million sequence reads (see Table 1). After removing adapter sequences, 17.5 million (99.4%) of the sequences were longer than 14 nt and 17.1 million (96.9%) of the reads could be mapped on the *N. gonorrhoeae* MS11 (V4) genome. Suboptimal read alignments and read alignments with more than three mismatches or indels were removed. The remaining 16.5 million reads yielded 23.2 million alignments (94.3%) that were used to generate read coverage tracks. Manual identification of transcriptional start and end sites (TSS and TES, respectively) was carried out by comparing TEX-treated with untreated read tracks thereby identifying 1172 primary TSS. Note that 46 secondary TSS were located downstream of primary TSS (Supplementary Table S1). In addition, 133 TSS were located within annotated CDS. Furthermore, 283 operons encoding a total of 771 protein coding genes were identified (Figure 1A). The operon with 10 ORFs, the highest number of CDS identified, coded for ribosomal proteins. The second largest was the ATP synthase operon consisting of 9 CDS. Four additional TSS were identified on the cryptic plasmid. The homology search for these four novel transcripts yielded a significant similarity to the hypothetical protein NGK_p0002, hypothetical protein NGK_p0003 and Amidophosphoribosyl transferase of NCCP11945.

**Table 1.** cDNA sequencing results, adapter removal and mapping overview

No. of reads	Illumina A -TEX	Illumina A +TEX	Illumina B -TEX	Illumina B +TEX	454 C -TEX	454 C +TEX	454 D -TEX	454 D +TEX
Sequenced	5 410 513	3 223 994	2 153 437	5 589 533	299 475	328 427	322 146	299 755
Processed	5 373 804	3 214 281	2 129 429	5 582 207	292 628	322 252	313 940	288 842
Mapped	5 318 864	3 209 065	2 084 169	5 576 139	280 501	315 114	89 925	206 705
Uniquely Mapped	2 484 119	2 887 125	925 437	5 489 994	169 728	200 189	45 887	98 145

Based on this TSS map, we calculated the length of 5′ leader sequences (5′ untranslated regions; 5′-UTR) for the 919 mRNAs with assigned primary TSS (Figure 1B). Leader sequences of the majority of mRNAs varied between 20 and 70 nt in length with a median of 54 nt. Leader sequences longer than 100 nt were found for 271 mRNAs; 32 leaders encompassed even more than 400 nt (Figure 1B). The by far longest leader with 4176 nucleotides was found for *sst*T, a serine/threonine transporter (locus id NGFG_01933; for further details see below). By contrast, NGFG_04253 (transposase), NGFG_01109 (hypothetical protein) and NGFG_00745 (Methylated DNA protein cysteine methyltransferase) are transcribed as leaderless mRNAs. The lengths of 3′-UTRs ranged from 0 up to almost 2000 nt, whereas the median was 57 nt and thus slightly larger than the median of 5′-UTRs (54 nt; Figure 1C).

A total of 93 genes were re-annotated, since their TSS is located downstream of the annotated translational start (Supplementary Table S2) including, for example, D-alanyl-D-alanine carboxypeptidase (NGFG_00246), ATP-dependent DNA helicase DinG (NGFG_01619) and a non-functional sigma factor σ54 (NGFG_01677) (26).

Genes with tandem promoters and two or more TSS have been identified for 62 genes. Out of these, 42 genes contained primary as well as secondary TSS and 23 genes were transcribed from a primary and an internal TSS. Examples for genes with primary and secondary TSS are a lipoprotein coding gene (NGFG_00049) and a zinc-binding alcohol dehydrogenase (NGFG_00324). For genes with primary as well as internal TSS, cytochrome c biogenesis protein (NGFG_00240) and disulfide bond formation protein B (NGFG_02168) are exemplary. Only gene NGFG_04218 encoding for a hypothetical protein was identified with primary, secondary and internal TSS (see Supplementary Table S2).

For each of the 2526 genomic regions on the chromosome, sense and antisense expression was quantified and normalized as RPKM values (reads per kilobase of gene model per million of mapped reads). The mean of sense expression was 1895 RPKM, while the mean of antisense expression was 91 RPKM (see Figure 1D). Thereby, sense expression is over 20 times as strong as antisense expression. Normalized expression within intergenic regions was slightly less than antisense expression (mean 75 RPKM). A genomic region was categorized as transcribed, if its RPKM value was greater than 41.7 RPKM. A number of 846 CDS were below this transcriptional threshold and 94 CDS even yielded less than 1.0 RPKM which were thus defined as not transcribed under these experimental conditions.

### Novel non-coding transcripts

**Figure 2.**
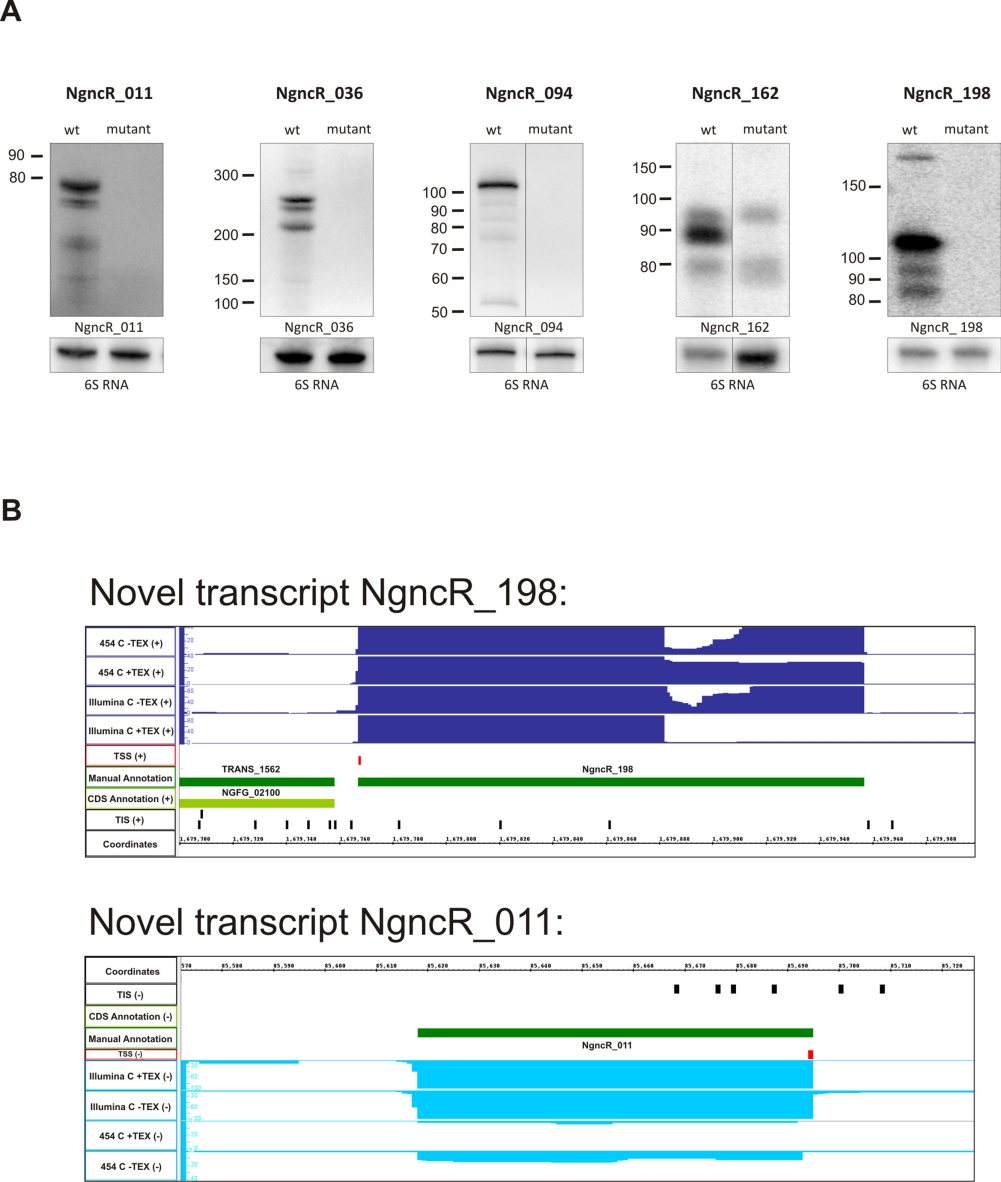
Novel transcripts and sRNA. **(A)** Northern blot validation of putative small RNAs. Expression of three new transcripts and two previously published sRNAs could be confirmed in MS11 strain. Wt MS11 as well as mutant strains were analyzed by northern blotting. Calculated RNA length were: 115 nt (short transcript) and 191 nt (long transcript) for NgncR_198, 88 nt for NgncR_162, 75 nt for NgncR_011, 239 nt for NgncR_036 and 109 nt for NgncR_094. Multiple bands visible for NgncR_198, NgncR_036 and NgncR_094 may have originated from degradation of the full-size products. **(B)** Visualization of sequence reads using the integrated genome browser for putative small RNA NgncR_011 and NgncR_198 obtained from non-treated and TEX-treated (+ TEX) libraries.

Additionally, we identified 253 novel transcripts without an existing CDS annotation (Supplementary Table S2). Of these non-coding transcripts, 59 mapped to intergenic regions, which is typical for small regulatory RNAs (sRNAs) (62–64). To unambiguously identify these non-coding RNAs, the nomenclature NgncR_001 – NgncR_253 (for *N. gonorrhoeae* non-coding RNA) was introduced, with the numbers in the transcript names corresponding to the genomic order. The transcription of 9 of these putative sRNAs was validated by northern blotting (Figure 2A, Supplementary Figure S1). Among the latter were also homologues of the previously published neisserial sRNAs Fur-regulated NrrF (NgncR_236)(29), σE sRNA (NgncR_011)(32) and the oxygen responsive FnrS/AniS (NgncR_094)(34). We created deletion mutants of the putative novel sRNAs NgncR_011, NgncR_036, NgncR_094, NgncR_162 and NgncR_198. By comparing northern blots of wild-type and deletion mutants the expression of these putative sRNAs was verified (Figure 2A). Exemplarily, the sequence reads for the two putative sRNAs NgncR_198 and NgncR_011 are depicted in Figure 2B.

### Antisense transcription

Strand-specific RNA-Seq approaches offer the opportunity to detect RNAs that are transcribed antisense to open reading frames (ORFs). We have discovered antisense transcripts for 194 genes that make up 16% of all primary transcripts detected. Gonococci contain a CDS of 5882 nucleotides that is a homologue of the *Bordetella pertussis* filamentous hemagglutinin (NGFG_01087; Supplementary Figure S2A). Despite a predicted promoter upstream of the start codon, expression of the protein has never been reported. Our RNA-Seq analysis indeed revealed a very strong promoter activity and a defined TSS, however, NGFG_01087 full size transcripts are rare. Two small and one larger antisense transcripts (NgncR_145) were detected suggesting a role of antisense RNA transcripts in regulation of NGFG_01087. Other interesting and unexpected cases for potential antisense regulation include the genes for Opa protein family. Nine of the 11 *opa* genes exhibit strong antisense transcription either as part of a long leader sequence of a gene transcribed antisense downstream of the *opa* gene (NGFG_04228, NGFG_04222) or as a non-coding transcript (NGFG_04296; NGFG_04295; NGFG_04209; NGFG_02238; NGFG_04124; NGFG_04090; NGFG_04076). The very long 755 bp leader sequence of gene NGFG_00699 (hypothetical protein, Supplementary Figure S2B) is antisense to the *opa* gene NGFG_04222. An example for an antisense regulation of an *opa* gene is given by the novel transcript NgncR_179 which overlaps with more than 250 bp antisense to *opa* gene NGFG_02238 (Supplementary Figure S2C).

Potential antisense regulation by very long leader sequences was also found for other genes. For example, the 515 nt leader of NGFG_00458 (4-hydroxybenzoate octaprenyltransferase) overlaps with 245 nt antisense to NgncR_035 and 31 bp antisense to NgncR_034. The latter transcript also reaches as antisense transcript NGFG_00456 (chorismate pyruvate lyase) upstream of NGFG_00458 (Supplementary Figure S2D). Since both proteins are part of the ubiquinone biosynthesis pathway it is possible that these transcripts antisense to leaders have regulatory functions.

### Phage clusters

**Figure 3.**
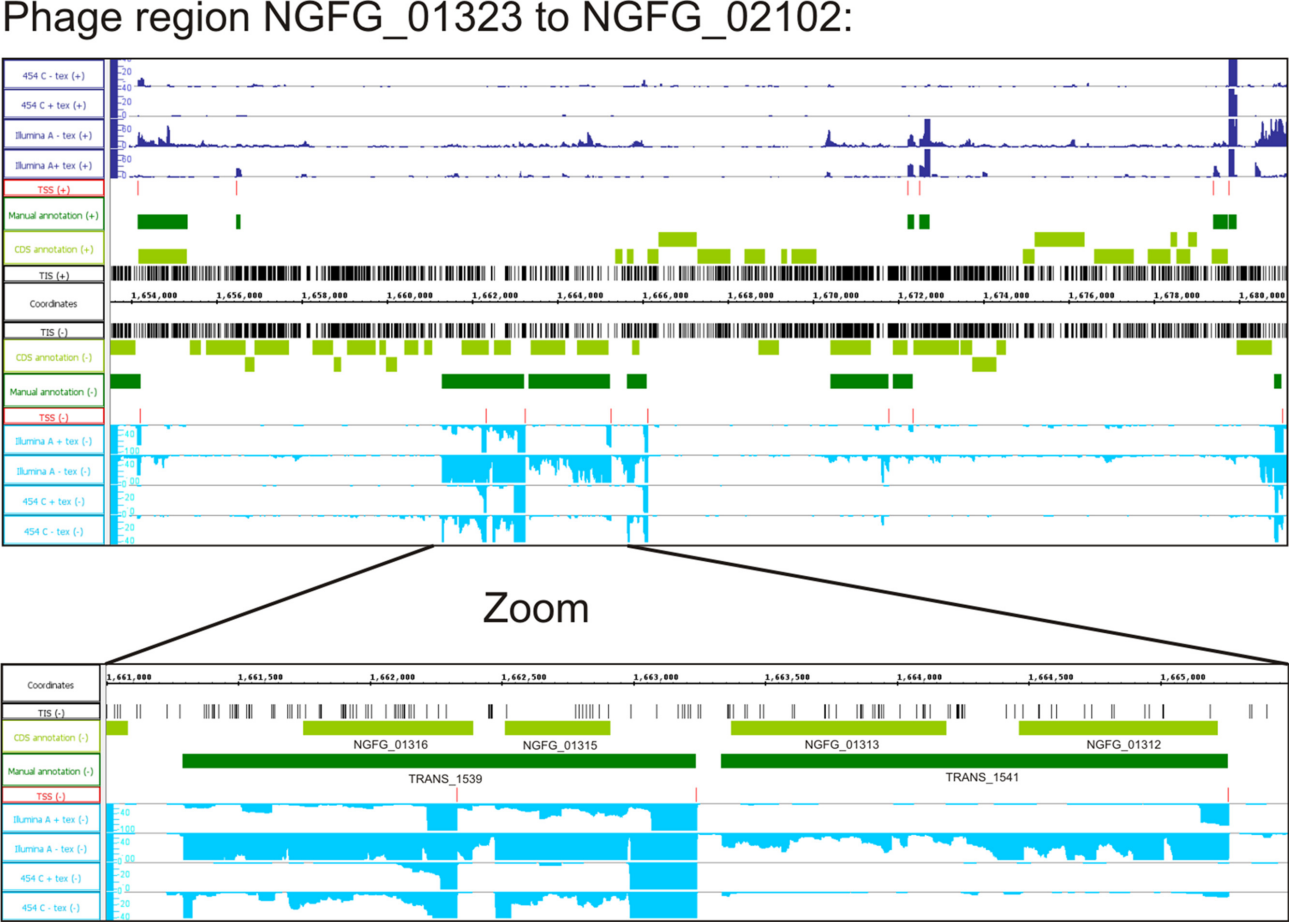
Phage gene cluster. Visualization of sequence reads of the RNA-Seq and TIS of the Tn-Seq using the integrated genome browser for the phage gene cluster NGFG_01323 - NGFG_02102 and a magnification of the transcriptionally active region including the phage repressor protein NGFG_01312.

At least five large clusters of phage-related genes are present in strain MS11 that share homology to phages previously described in strain FA1090 (65) (NGFG_00618 - NGFG_01272 of 43.8 kbp homologous to NgoΦ1; NGFG_00651 - NGFG_02078 of 41.9 kbp homologous to NgoΦ2; NGFG_01323 - NGFG_02102 of 26.6 kbp homologous to NgoΦ3; NGFG_04316 - NGFG_00715 of 13.3 kbp homologous to NgoΦ4; NGFG_01070 - NGFG_01054 of 10.2 kbp homologous to NgoΦ5). With the exception of several hot spots, these genomic regions were mainly transcriptionally silent (Figure 3). Some of the active genes code for homologues of phage repressor proteins (e.g. gene NGFG_01312; Figure 3) involved in maintenance of phage lysogeny.

### Riboswitch detection

Riboswitches are defined regions of mRNAs to which certain metabolites bind to alter transcription or translation. So far, no riboswitches have been identified in *Neisseria*. A genome-wide riboswitch analysis based on the 26 riboswitch families annotated in the Rfam 11.0 database revealed six hits on the ring chromosome and no hits on the plasmid (see Supplementary Table S3). All six hits were located in the 5′-UTR of protein-coding genes, all in a maximal distance of 12 bp adjacent to the annotated transcription start sites. While for some riboswitches the motif start was identified even upstream of TSS, all conserved and structure forming nucleotides were downstream of the TSS as expected. The 5′-UTRs of the six identified riboswitch regulated genes ranged between 47 and 303 bp with a median of 180.5 bp and thus are notably longer than the average 5′-UTR with a median of 54 bp.

Using this approach, highly significant candidates for the widespread thiamine pyrophosphate responsive riboswitches (66) (NGFG_01842; NGFG_01876), a glycine family riboswitch (67) (NGFG_01512) and a riboswitch involved in the biosynthesis of queuosine (68) (NGFG_02134) have been identified. Another queuosine riboswitches (NGFG_00267) and a riboswitch for S- adenosylmethionine (69)(NGFG_00245) were also predicted but did not reach significance.

### Gonococcal promoters frequently contain DUS

Next we conducted a genome-wide promoter analysis using motif and similarity network approaches. We focused on promoter regions of protein coding genes with manually annotated primary TSS. Regions spanning 70 nt upstream of 890 annotated TSS have been extracted from the MS11 genome yielding a total of 58 995 bases of putative promoter regions and comprising 2.645% of the entire genome.

**Figure 4.**
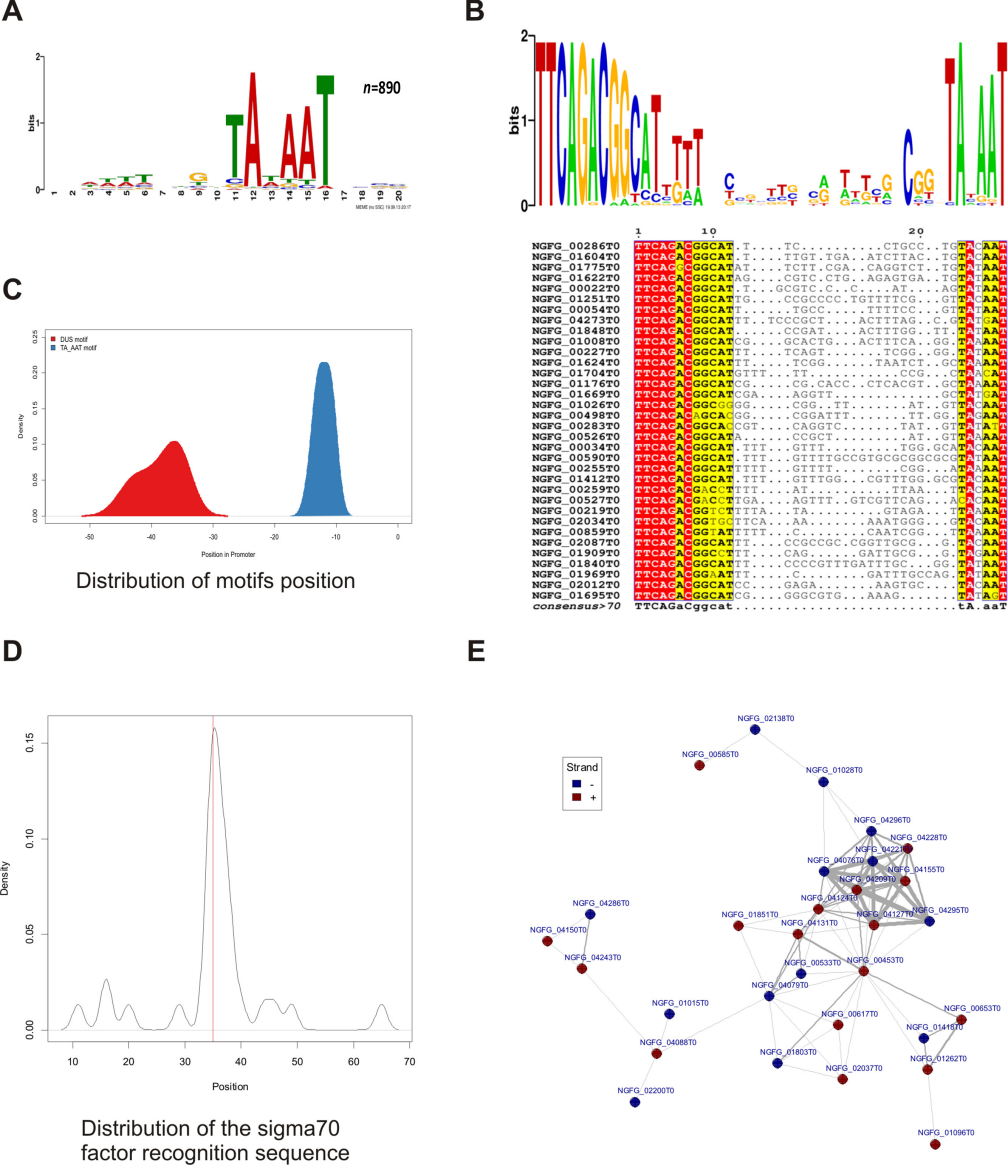
Promoter analysis and motif search. **(A)** Genome-wide motif analysis of promoter regions identified a TATA box-like motif in all promoters. **(B)** Multiple sequence alignment and sequence logo of the DUS and the TATA-box motifs including the in-between sequence region of 34 promoters where the DUS motif is anchored around the −35 position. **(C)** Positional distribution of the DUS and the TATA-box like motif in these sequences. **(D)** A conserved TTGACA motif was identified by exact matching around the position −35 in 30 promoter sequences. **(E)** Network analysis of highly similar promoter sequences reveals a heterogeneous largest component of 30 promoters including densely clustering opacity proteins. Nodes in the network represent promoters and edges depict significant similarity between the promoter sequences; stronger edges represent higher similarity scores.

In a classical motif analysis we identified enriched sequences in the promoter regions. As expected the most abundant motif was the Pribnow box, which was detected in all 890 promoters (Figure 4A). A classical TTGACA −35 box sequence was found in only 30 promoter sequences (Figure 4D), suggesting that an *E. coli*-like σ70 promoter is absent in most of the protein coding genes in gonococci (Figure 4C). The second-most abundant motif was the DUS TTCAGACGGCAT with 170 occurrences in the promoter regions. Relative to the transcriptional direction of the corresponding gene, 101 DUS sequences were identified in forward and 69 in reverse orientation. This denotes a weak orientation bias toward the transcriptional direction of the related gene (*P*-value = 0.0122). In 34 promoter regions out of those with a DUS motif in forward direction, the DUS is located within a window of 10 bases around the −35 position. A multiple sequence alignment of these promoters shows a highly conserved DUS motif upstream of a highly conserved Pribnow box (Figure 4B). The positional distribution of both motifs in these sequences with the central DUS is depicted in Figure 4C. Approximately a third of the DUS motifs were located inside of the annotated terminator regions, which have been described to be associated to DUS motifs. However, more than 70% of identified DUS motifs were positioned inside promoter relevant regions. Another motif identified by these analyses contained the consensus sequence of a classical RpoH promoter (Supplementary Figure S3A) that has been identified for other gonococcal genes before (70).

To identify potentially co-regulated genes we performed a comprehensive sequence similarity analysis of all promoter regions, followed by network analysis of the resulting promoter clusters. All-against-all pairwise sequence comparisons of all promoter regions yielded a similarity matrix based on the scores of 395 605 alignments. Furthermore, we generated a set of random sequences with the same base composition as the observed promoter region, to contrast the observed alignment scores against the randomized background distribution (Supplementary Figure S3C). Using a score threshold based on this background distribution we constructed a promoter similarity network consisting of 138 promoters and 173 significant similarity relations between them. The largest component of this network comprises 30 promoters with 85 high similarity relationships. Among others, this cluster includes 11 hypothetical proteins and a tightly connected cluster of 9 opacity proteins (Figure 4E). The second largest component comprises 7 promoters and 15 edges, whereas the third cluster of 5 genes includes ComEA proteins and a loosely linked queuine tRNA-ribosyltransferase (NGFG_00439). The remaining network clusters into small components of promoter pairs and triplets.

### Identification of essential genes

To define the core of essential genes in GC we established a genome-wide random transposon mutant library in *N. gonorrhoeae* MS11 (Supplementary Figure S4). Chromosomal DNA from GC was subjected to *in vitro* Tn5 mutagenesis, mutated DNA was transformed into GC and recombinant bacteria were selected for kanamycin resistance. More than 100 000 individual colonies were obtained and subsequently pooled (Supplementary Figure S4A). Genomic DNA from this pool was extracted, fragmented and ligated to Illumina PE adaptors (see Materials and Methods for details). Selective amplification of the genomic sequences adjacent to TIS was performed by PCR and the products were sequenced on a Illumina HiSeq-2000 next-generation sequencing platform using a custom sequencing primer that binds to the transposon mosaic end (Supplementary Figure S4B). Thus, the location of the TIS can be identified with single nucleotide resolution. Two different sequence runs yielded 84 335 (Library A) and 86 327 (Library B) TIS (Table 2). The intersection set of libraries A and B consisted of 57 840 TIS resulting in 112 822 distinct TIS. The highly correlated (Pearson's correlation coefficient *ρ* = 0.994; *P*-value < 2.2e-16) TIS sets of libraries A and B were merged to one combined TIS data set for further analyses.

**Table 2.** Tn sequencing, processing and mapping results

No. of reads	A	B	A + B
Sequenced	54 346 653	30 541 365	84 888 018
Processed	38 534 885	17 804 632	56 339 517
Mapped	36 258 405	16 360 608	52 619 013
Unique TIS	84 335	86 327	112 822

**Figure 5.**
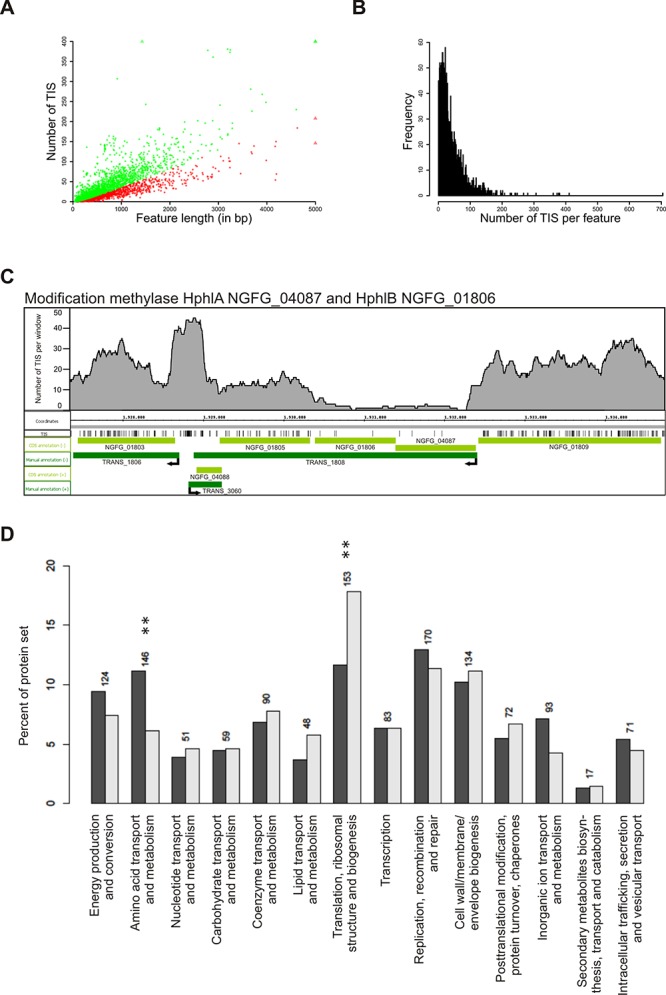
Tn-Seq analysis. **(A)** Scatterplot of TIS counts versus gene length. Red dots show genes with significantly lower TIS counts while green dots represent genes which are not significant. Triangles symbolize dots outside the borders of the plot. **(B)** Distribution of TIS counts per gene. **(C)** Distribution of identified TIS (black vertical lines) on a segment of the *N. gonorrhoeae* MS11 genome. For each position, the number of TIS in range of 200 nt in both directions was plotted. CDS annotations (light green) and transcript annotations (dark green) on plus and minus strands were visualized. The non-essential genes NGFG_01803, NGFG_ 04088 and NGFG_01809 have dense TIS, while essential modification methylases HphIA (NGFG_04087) and HphIB (NGFG_01806) display very few insertions and thus constitute essential genes. The potential promoter region of TRANS_1808 (modification methylases HphIA and HphIB) upstream of NGFG_04087 shows no TIS and, therefore, seems to be essential as well. **(D)** Relative COG class distribution of all CDS (dark gray) and of essential CDS (light gray). Significantly enriched or depleted COG classes are marked with stars.

For each of the 2576 genomic regions containing protein-coding, non-coding and novel transcripts the number of distinct TIS is shown in Supplementary Table S1. The number of TIS per gene varied between 0 and 705, with a median of 29 (Figure 5B). The TIS numbers are correlated to the length of the genomic regions (Pearson's correlation coefficient 0.70, *P*-value < 2.2e-16; see Figure 5A), demonstrating that the TIS were uniformly distributed over the normalized length of the genomic regions with a slightly higher frequency at the 5′- and 3′-ends of the significantly ‘essential’ genomic regions (see Supplementary Figure S5A).

We assigned *P*-values for gene essentiality assuming uniform transposon insertion rates across the whole genome and neutral fitness costs (Supplementary Figure S4C) (71). Based on this test, 827 genes were found to be significantly depleted in TIS (*P*-values < 0.05) out of which 286 genes are in operon structures and 230 genes showed low expression levels (see Supplementary Figure S5B). Since transposon insertions in a non-essential gene might disrupt the expression of an essential gene downstream within the same operon, we excluded genes in operons to avoid identification of false positives. Additionally, we included the expression information gained by RNA-Seq (see above), since expression of a gene is mandatory for defining it as essential. In total 353 of the 2562 genomic features on the ring chromosome that were not arranged in an operon showed significant underrepresentation of TIS in combination with normalized expression values of more than 41.7 RPKM (Supplementary Figure S5B). These were designated as essential for GC survival and growth (Supplementary Table S4). These include, for example, the methyltransferases (HphlA, NGFG_04087 and HphlB, NGFG_01806; Figure 5C), which play a role in restriction modification. Only very few TIS were detected in the coding region of NGFG_04087 and NGFG_01806 (*P* = 0.00061 for both; Figure 5C). By contrast, the genes upstream and downstream of the operon comprising NGFG_04087 and NGFG_01806, NGFG_01809 and NGFG_01803, display high TIS density which results in *P*-values of 1 and 0.99, respectively, thereby defining NGFG_01809 and NGFG_01803 as non-essential genes (Figure 5C). Furthermore, all aminoacyl-tRNA synthetase genes and seven out of the eight subunits of the multimeric DNA polymerase III were identified as essential (Supplementary Table S4). The remaining gene NGFG_00714 (epsilon subunit, *P* = 1) was unlikely to be essential. Interestingly, NGFG_00762 (*P* = 0.00061) also encodes an epsilon subunit and thus might functionally replace NGFG_00714. Our study also confirmed several genes (*P* < 0.01) that have been previously identified as essential: Omp85 (NGFG_01715, *P* = 0.00061) (72), PorB (NGFG_01725, *P* = 0.00061) (73) and the alternative sigma factor RpoH (NGFG_00430, *P* = 0.00636) (21).

Many of the predicted essential genes are required for fundamental biological processes, including amino acid transport, DNA replication, translation and cell wall biosynthesis (Figure 5D). Interestingly, up to 16.3% (135 genes) of the predicted essential genes are hypothetical proteins. We validated essentiality for a subset of these genes. For instance, we performed a genetic footprinting assay (56–59) in the genomic region of NGFG_01048–01053 (736587–732215). The uncharacterized gene NGFG_01049 (*P* = 0.00061) as well as NGFG_01051 encoding ferredoxin-NADP^+^ reductase (*P* = 0.00061) are predicted essential genes based on their *P*-values. PCR products corresponding to an insertion in these genes *in vivo* were rarely detected on an agarose gel when compared to the PCR products obtained from *in vitro* template. However, PCR products corresponding to an insertion in the surrounding non-essential genes were detected. These data are in agreement with the insertion patterns detected in Tn-Seq (Supplementary Figure S6A) and illustrate that NGFG_01049 and NGFG_01051 are indeed essential in *N. gonorrhoeae*. Conditional knockout assays were performed to test essentiality of other putative essential genes. The predicted promoter sequence 117 bp upstream of a transcript encoding the 50S ribosomal proteins L35 and L20 (NGFG_00442 and NGFG_00443) was exchanged by a Kanamycin-P_trc_ cassette. The P_trc_ promoter is IPTG-inducible as lacI^q^ can bind there as a repressor. In the recombinant strains the expression of the genes of interest thus can be selectively inhibited by omitting IPTG. The Ptrc promoter mutants NGFG_00442–00443 are able to grow on GC-plates supplemented with 0.5 mM IPTG but show growth defects when IPTG is omitted. These data indicate that the ribosomal protein-encoding genes NGFG_00442 and NGFG_00443 (*P* = 0.00061 and 0.00245, respectively) are essential for GC growth (Supplementary Figure S6B).

In addition to conditional knockout assays and DNA footprinting analysis, several putative essential genes were validated by monitoring the growth of gonococci after deletion of the respective genes. Bacteria were not viable as soon as genes like PorB (NGFG_01725, *P* = 0.00061), the beta-ketoacyl acyl-carrier protein synthase II (NGFG_01674, *P* = 0.00061) or the uridylate_kinase (NGFG_01912, *P* = 0.00111) were deleted. In parallel, knockout experiments were performed of non-essential genes like the FKBP-type peptidyl-prolyl cis-trans isomerase FkpA (NGFG_02032, *P* = 0.8841), a hypothetical protein NGFG_01393 (*P* = 1) and an ABC-transporter ATP-binding protein/permease (NGFG_01643, *P* = 1) and these mutants were viable (data not shown).

## DISCUSSION

The recent emergence of multi-resistant strains of GC calls for the development of new anti-gonococcal therapies. A straightforward approach is the identification of new gonococcal target proteins that fulfill essential functions and are thus potential targets for anti-gonococcal drugs. A classical common approach was the *in silico* prediction of metabolic pathways based on the genome sequences as well as transcriptome and proteome analysis that helped to identify potential targets. In contrast to this predictive approach, we have used a combination of dRNA- and Tn-Seq to investigate the detailed gene structure and the set of essential genes in *N. gonorrhoeae*. The experimental identification of the gonococcal core gene set unveiled numerous new genes without known function as well as previously described essential genes.

We have applied dRNA-Seq which comprises the enzymatic depletion of RNAs with 5′ monophosphates to define the TSS of gonococcal genes to the single nucleotide level. As expected, most of the TSS are congruent with the annotated CDS. In addition, 253 mainly newly identified transcripts were located either antisense to predicted CDS or in intergenic regions indicating that these transcripts are part of a yet undiscovered level of gene regulation in gonococci. However, many of the annotated transcripts could not be detected in our sequences despite the high depth of sequencing. These genes may constitute pseudo-genes or they are either expressed at very low levels or under different growth conditions. We also did not expect to find TSS for all annotated genes since, despite the highly dynamic nature of the gonococcal genome, genes are also organized in operons in *N. gonorrhoeae.* The operon structure can be predicted by comparing reads derived from total RNA with those from enriched TEX-treated RNAs. In enriched libraries, TSS are detected only for the first gene of the operon. This approach allowed for the first time the definition of 283 operons in gonococci yet with an average gene number of only 2.7.

Of the novel non-coding transcripts, 59 were found in intergenic regions and very likely belong to the class of regulatory sRNA including the previously characterized iron-regulated NrrF (30) or FnrS that is upregulated under oxygen limitation (34). Since target prediction for bacterial sRNAs is still difficult (74), the role of this large number of regulatory RNAs is still unknown. GC belongs to the class of bacteria that express the conserved RNA-binding protein Hfq involved in sRNA-mediated regulation of mRNA stability and translation (75). Deletion of Hfq in gonococci altered the mRNA levels of 369 CDS (76), pointing to a profound and global role of neisserial sRNAs in gene regulation.

An entirely unexpected finding was the strong antisense transcription of 9 of the 11 *opa* genes. All *opa* genes are constitutively transcribed from strong promoters but most of these genes are out of frame due to changes in pentameric repeats (CTCTT) within the leader peptide encoding sequence (77). Belland *et al.* previously described an incomplete synthesis of *opa* mRNA from out-of-frame genes and speculated on a *rho-*dependent downregulation of non-translated *opa* RNAs (78). However, a non-translated *pilE* RNA was not degraded in these strains (78), indicating that turnover of non-translated *opa* RNAs differs from that of other non-translated RNAs. Since we found no evidence for antisense transcription in *pilE* mRNA, antisense control could be the mechanisms how GC controls mRNA levels of phase variable out-of-frame *opa* genes. In this model, non-translated *opa m*RNA may bind to the antisense transcripts and be degraded by ribonuclease III. Translation of in-frame *opa* mRNA may interfere with binding of antisense transcripts and thereby prevent degradation. This mechanism would ensure that the vast majority of useless out-of-frame *opa* mRNAs are immediately recycled and do not accumulate.

The precise definition of TSS by sequencing enriched libraries enabled us to search for motifs 70 bp upstream of the transcription start. A classical RpoD (σ70) -10 consensus sequence motif (TAHAAT) was identified for 890 promoters of coding genes. Most of these genes, however, missed a classical −35 box (TTGACA) which was only detected for 30 genes. Interestingly, the DUS was identified upstream of 170 CDS. Out of these 34 promoters contained an inverted DUS at the −35/−37 region that could constitute a classical −35 RpoD box with two point mutations (TTCAGA). It is interesting to speculate that the DUS may have evolved as part of the classical gonococcal promoter. The position precisely upstream of the CDS would ensure the uptake and processing of potentially active gonococcal genes.

We further generated a complex Tn5 transposon library and established an Illumina-based Tn-Seq method to define the core of essential genes in gonococci. Due to the very high sequence coverage TIS were detected in nearly all genes although with extremely different frequencies. Only the application of statistical analysis allowed us to predict unequivocally the genes with significant underrepresented TIS which represent genes that support gonococcal fitness in rich medium. The identification of these essential genes is particularly important since GC acquired multiple antibiotics resistances over the last decades and essential gene products are excellent targets for new antibiotics. The approach chosen here provided a large list of potential anti-gonococcal targets some of which have an enzymatic function and are thus potentially druggable. In addition, development of drugs that target more than one of these essential gene products will significantly reduce the occurrence of new antibiotic resistances. Furthermore, the comprehensive transcriptomic data together with this list of essential genes is also a valuable source to better understand the physiology of this important pathogen since information about the expression and essentiality is provided for each single gene.

Besides a direct crucial role for the fitness of gonococci, some of these factors may affect viability in a more indirect way. For example, essential genes NGFG_00630 (homologous to NGO_0479 of NgoΦ1 in FA1090 (65)) and NGFG_02188 (homologous to NGO_1116 of NgoΦ2 in FA1090 (65)) are homologues of the lambda repressor cI (65) and may reactivate a lysogenic phage upon inactivation. Another interesting example is provided by the NGFG_00971 gene that originally has been annotated as hypothetical protein. A scan with the RASTA-Bacteria prediction tool (Rapid Automated Scan for Toxins and Antitoxins in Bacteria, (79)) identified NGFG_00971 as anti-toxin. Inactivation of NGFG_00971 may release the toxic activity of NGFG_00972 and thus cause direct killing of mutated derivatives rather than affecting gonococcal fitness.

We applied additional criteria to further subdivide the large list of genes with significantly underrepresented Tn5 insertions. The top 353 essential genes are neither organized in operons and, based on our dRNA-Seq data, are all expressed under the conditions chosen to generate the library. Genes with underrepresented Tn5 insertions that are organized in operons may have an essential function but it is also possible that the Tn5 integrations affect the expression of downstream genes with essential function. Likewise, potentially essential genes with no significant expression at the time of analysis may be expressed under different conditions and thus provide a fitness advantage. Clearly, not all the genes identified as essential are indispensable for gonococcal survival. For example, Hfq has been identified by us as essential with a very high significance although deletion mutants are viable with a strongly reduced fitness though (71). Many other genes identified by our approach are well-known to be involved in housekeeping functions like translation, DNA replication and division (Figure 5D). Furthermore, proteins like, e.g. Omp85 (72), the PorB porin (73,80) and the sigma factor RpoH (21) are known to be indispensable for gonococcal survival and were identified with high significance. Genetic footprinting experiments and knockout studies with several thus far unknown essential gonococcal genes demonstrated in addition the robustness of our Tn-Seq approach.

In summary, we provide here the first comprehensive analysis of the TSS and the definition of essential genes of the major pathogen *N. gonorrhoeae*. The applied strategy has several advantages since the combined definition of the gene structure and activity together with its function provides a new quality of information. Our basic analyses focused on growth conditions on rich medium, however, the full potential of the approach will be exploited in future by investigating different infection conditions. This new insight into the regulation and function of disease-related genes will hopefully help to develop effective strategies to combat this highly adapted human pathogen.

## SUPPLEMENTARY DATA

Supplementary Data are available at NAR Online.

SUPPLEMENTARY DATA
