# Supplementary material for: Transcriptional landscape and essential genes of Neisseria gonorrhoeae
Source: Nucleic Acids Res. 2014 Aug 20;42(16):10579–95. doi: 10.1093/nar/gku762 (PMC4176332; doi:10.1093/nar/gku762)
Supplement: SUPPLEMENTARY DATA [file supp_42_16_10579__index.html]

Transcriptional landscape and essential genes of Neisseria gonorrhoeae — Transcriptional landscape and essential genes of Neisseria gonorrhoeae — SUPPLEMENTARY DATA 

# Transcriptional landscape and essential genes of *Neisseria gonorrhoeae*

## SUPPLEMENTARY DATA

**Files in this Data Supplement:**

- SUPPLEMENTARY DATA
