# Supplementary material for: Transcriptional landscape and essential genes of Neisseria gonorrhoeae
Source: Nucleic Acids Res. 2014 Aug 20;42(16):10579–95. doi: 10.1093/nar/gku762 (PMC4176332; doi:10.1093/nar/gku762)
Supplement: SUPPLEMENTARY DATA [file supp_gku762_nar-01255-z-2014-File007.zip › NAR-01255-Z-2014.R1 Suppl files/Supplemental_material_Remmele_et_al.docx]

# Supplemental material

## Supplemental tables

### Table S1: Table with CDS, transcript, TSS, TIS, expression and homology information

For all genomic features, this table gives information about the location and function of CDS, the type (1: primary, 2: secondary, 3: internal) and location of identified transcription start and end sites, antisense overlap, the normalized sense and antisense expression, the number of TIS and the significance of TIS under-representation, as well as homology information to FA1090 and NCCP11945.

**Table S2: Novel transcripts and transcripts with primary, secondary and internal TSS**

This table groups the genomic features into the categories: novel transcripts, genes with internal TSS but without primary TSS, genes with primary and secondary TSS, as well as genes with primary and internal TSS.

**Table S3:** **Overview of the detected riboswitches in MS11**

The columns contain information about the detected riboswitch family, the e-value, genomic position, as well as the relative position in the transcript, the ID, name and genomic position of regulated coding sequences.

### Table S4: Core essential genes

For genomic features which are defined as core essential, this table gives information about the location and function of CDS, the type (1: primary, 2: secondary, 3: internal) and location of identified transcription start and end sites, antisense overlap, the normalized sense and antisense expression, the number of TIS and the significance of TIS under-representation, as well as homology information to FA1090 and NCCP11945.

### Table S5: Oligonucleotides

| **Name** | **Oligonucleotide Sequence (5’-3’)** |
| --- | --- |
| Adaptor sense | p-GATCGGAAGAGCGGTTCAGCAGGAATGCCGAG |
| Adaptor antisense | ACACTCTTTCCCTACACGACGCTCTTCCGATC*T |
| TnSeq-PE-Index2 (library A) | CAAGCAGAAGACGGCATACGAGAT**ACATCG**CGGTCTCGGCATTCCTGCTGAACCGCTCTTCCGATC |
| TnSeq-PE-Index3 (library B) | CAAGCAGAAGACGGCATACGAGAT**GCCTAA**CGGTCTCGGCATTCCTGCTGAACCGCTCTTCCGATC |
| P5-ME | biotin-AATGATACGGCGACCACCGAGATCTACGGTTGAGATGTGTATAA GAGACAG |
| TnSeq Primer | ACCGAGATCTACGGTTGAGATGTGTATAAGAGACAG |
| TnSeq index SP | GATCGGAAGAGCGGTTCAGCAGGAATGCCGAGACCG |
| Tn ME sequence | GGTTGAGATGTGTATAAGAGACAG |
| 01048-up-f | GCCGTCTGAAcagccgattcatagacgaaatgcc |
| 01052-down-r | CTTCGTATGCTTGGCGGTGGC |
| NgncR_011_up_s | GCCGTCTGAACATCGTTGCGGATAACCAGC |
| NgncR_011_up_as | TGAGACACAATTCATCGATGATGCCGTCGTAAAAGCTCAAAAAACATTTGC |
| NgncR_011_down_s | TGCAGGCATGCAAGCTTCAGACGGACGGCAAGCAGTTAAATAGATTTT |
| NgncR_011_down_as | CGGCGATAAATGCTTTTGCCTGTCCGCCG |
| NgncR_036_up_s | GCCGTCTGAAGGTTTTGGAAATGCCGTCTG |
| NgncR_036_up_as | TGAGACACAATTCATCGATGATAATGCCGTCTGAAGTCCTGC |
| NgncR_036_down_s | TGCAGGCATGCAAGCTTCAGGTTTTGCTGTTGATGGAAGCATGATTGT |
| NgncR_036_down_as | TCCCGTTCGCGGCATTTACCAC |
| NgncR_094_up_s | GCCGTCTGAAAGCTGCCGACGATTTGATTGAACGCATCC |
| NgncR_094_up_as | TGAGACACAATTCATCGATGATTCAAACAAAAACCGTTATCGAG |
| NgncR_094_down_s | TGCAGGCATGCAAGCTTCAGGGCTTTTATGTGAAGTAAAATCCG |
| NgncR_094_down_as | CAGACGGCACTTGTCGGGTATCGGCGG |
| NgncR_162_up_s | GCCGTCTGAAGGCGATTTGTCCGCACAATG |
| NgncR_162_up_as | TGAGACACAATTCATCGATGATCCGCCGTTTCTTATCTGCTG |
| NgncR_162_down_s | TGCAGGCATGCAAGCTTCAGGGATGTGTGCATTTTTTATCTCCGC |
| NgncR_162_down_as | CCCGATATGTCTGCCTATCGGGA |
| NgncR_198_up_s | GCCGTCTGAAAATAGCCGCCTGTCATCGTC |
| NgncR_198_up_as | TGAGACACAATTCATCGATGATGGGAACGGTTTTATTCTAGTACAG |
| NgncR_198_down_s | TGCAGGCATGCAAGCTTCAGAATGCCGTCCGAACCTTCAG |
| NgncR_198_down_as | CTGCCCGAAGCCGTCGCCTAC |
| Kan-cassette_s | ATCATCGATGAATTGTGTCTCAAAATCTCTGAT |
| Kan- cassette _as | CTGAAGCTTGCATGCCTGCA |
| Kan-SpeI-F | CGACTAGTATCATCGATGAATTGTGTCTC |
| Kan-SacI-R | TAGAGCTCCTGAAGCTTGCATGCCTG |
| Ptrc-F | GCGCCGACATCATAACGGTTCTG |
| Ptrc-R | CATGGTCTGTTTCCTGTGTGAAATTG |
| Kan-cassette-R | CTGAAGCTTGCATGCCTGCA |
| rib-up-f | GTGCGTTTAATCAGTGAGTCAGGC- |
| rib-up-r | TGCAGGCATGCAAGCTTCAGGCAATCGGAGTAAGCGGAAAA |
| rib-down-f | CACACAGGAAACAGACCATGCCTAAAATGAAAACCAAGTCTAGCG |
| rib-down-r | CGGCTTTATCGAACACGGCC |

p: phosphorylation; *: phosphothioate bond; biotin: biotin-TEG modification; the sequences in bold are the barcodes; the underlined sequences are DUS (*Neisseria* DNA Uptake Sequence).

### Oligos for Northern blot detection of putative small RNAs

| **Target** | **Oligonucleotide Sequence (5’-3’)** |
| --- | --- |
| NgncR_011 | GGAGGCTTCCCAAGGAGTATTGAT |
| NgncR_036 | TGTCGGTTGGGAGAATTCTTAATT - |
| NgncR_052 | TAAAAATTCATATCCTTCGGGCGT |
| NgncR_094 | AGGTAAAATCAAGACGGATTGGGA |
| NgncR_130 | GGGCTTGTACAGGGAGTAGGAAAA |
| NgncR_162 | CGGTTGAATGTGTGCCAAGTCTA |
| NgncR_163 | TGTGTGCCAAGTCTACAAAGGAGA |
| NgncR_198 | CCCATATTTCGGAACAGTCTTGC |
| NgncR_236 | ATGTCTCGTATATGCCGACTCCAA |
| 6S RNA | GGCAACCTTAAGCGAACTTATTGG |

## Supplemental figure legends

**Figure S1:** Northern blot validation of putative small RNAs. Expression of 3 new transcripts and 1 previously published sRNA was confirmed in MS11 strain by Northern blotting. Calculated RNA length were: 80 nt for NgncR_052, 148 nt for NgncR_130, 90 nt for NgncR_163, 153 nt for NgncR_236 (NrrF).

**Figure S2:** Visualization of sequence reads of the RNA-Seq and TIS of the Tn-Seq using the integrated genome browser. (A) Locus of the gonococcal filamentous hemagglutinin homolog (NGFG_01087). (B) NGFG_00699 (hypothetical protein) with long leader sequence. (C) Locus of the opa gene NGFG_02238. (D) Locus of the ubiquinone biosynthesis cluster.

**Figure S3**: **(A)** Multiple sequence alignment and sequence logo of RpoH-like motif identified in 10 upstream regions of a classical TAHAAT box. **(B)** Base frequencies in promoter sequences (red) compared to overall genome composition (blue) reveals a compositional bias toward (A and T). **(C)** Distribution of the observed alignment scores (Signal) compared to the distribution of randomized alignment scores (Noise) shows an enrichment of high scores in the GC promoter data.

**Figure S4**: Generation and sequencing of a transposon mutagenesis library. **(A)** Construction of a genome-wide random transposon mutagenesis library. *Neisseria* genomic DNA is transposed *in vitro* by Tn5 transposon containing a kanamycin resistance marker, and then transformed naturally into a *Neisseria* population. The bacteria are selected on GC agar plates with kanamycin and subsequently pooled. **(B)** Preparation of Illumina sequencing sample. Genomic DNA from this pool is extracted, fragmented and ligated to Illumina PE adaptors. Transposon junctions are amplified with the primers complementary to transposon mosaic ends (ME) and adaptors. The DNA fragments carrying terminals (orange, blue) compatible to the Illumina flow-cell are sequenced specifically with the sequencing primer (Tn-Seq Primer) that binds to transposon mosaic end and the library specific barcode is sequenced by Tn-Seq index SP. **(C)** Basic steps for TN-Seq analysis: Short reads of transposon mutagenesis libraries were curated by removing Illumina adapter sequences as well as reads with too less nucleotides. Curated short reads were mapped onto the reference sequences (ring chromosome and plasmid). Transposon insertion sites were identified via genomic start positions of the mapped reads. For p-value assignment, a permutation test with 10,000 permutation cycles was performed.

**Figure S5**: **(A)** Relative TIS frequencies per percentile of gene length for essential genes (red) and non-essential genes (green). **(B)** Bar plot displaying the number of genes assigned to the eight groups according to the factors: significance of TIS under-representation, expression and transcript structure.

**Figure S6:** **(A)** Essential genes were validated by genetic footprinting. DNA fragments containing predicted essential genes from *in vitro* and *in vivo* libraries were amplified with transposon-specific primer (Tn ME sequence) and a gene region specific primer. The left image shows the genetic footprint analyzed in the 5’ direction and the right image from the 3’ direction. Lane 1, analysis of *in vitro* libraries; lane 2, from *in vivo* libraries; M, DNA marker. Grey: non-essential genes; green: essential genes required for *Neisseria* growth or survival. **(B)** Growth phenotypes of *N. gonorrhoeae* MS11 wild type or NGFG_00442-00443 conditional knockout mutants (two different colonies) on GC agar plates in presence of (right) or absence of (left) 0.5 mM IPTG. The gonococci were grown on GC agar plate with IPTG for 16-20 h at 37°C in 5% CO_2_ in a humidified atmosphere and then collected in PPM medium. Approximately 10^7^ gonococci were streaked on GC agar plates with or without IPTG and 24 h later the phenotypes were recorded.
