# Supplementary figures and images for: Transcriptional landscape and essential genes of Neisseria gonorrhoeae
Source: Nucleic Acids Res. 2014 Aug 20;42(16):10579–95. doi: 10.1093/nar/gku762 (PMC4176332; doi:10.1093/nar/gku762)

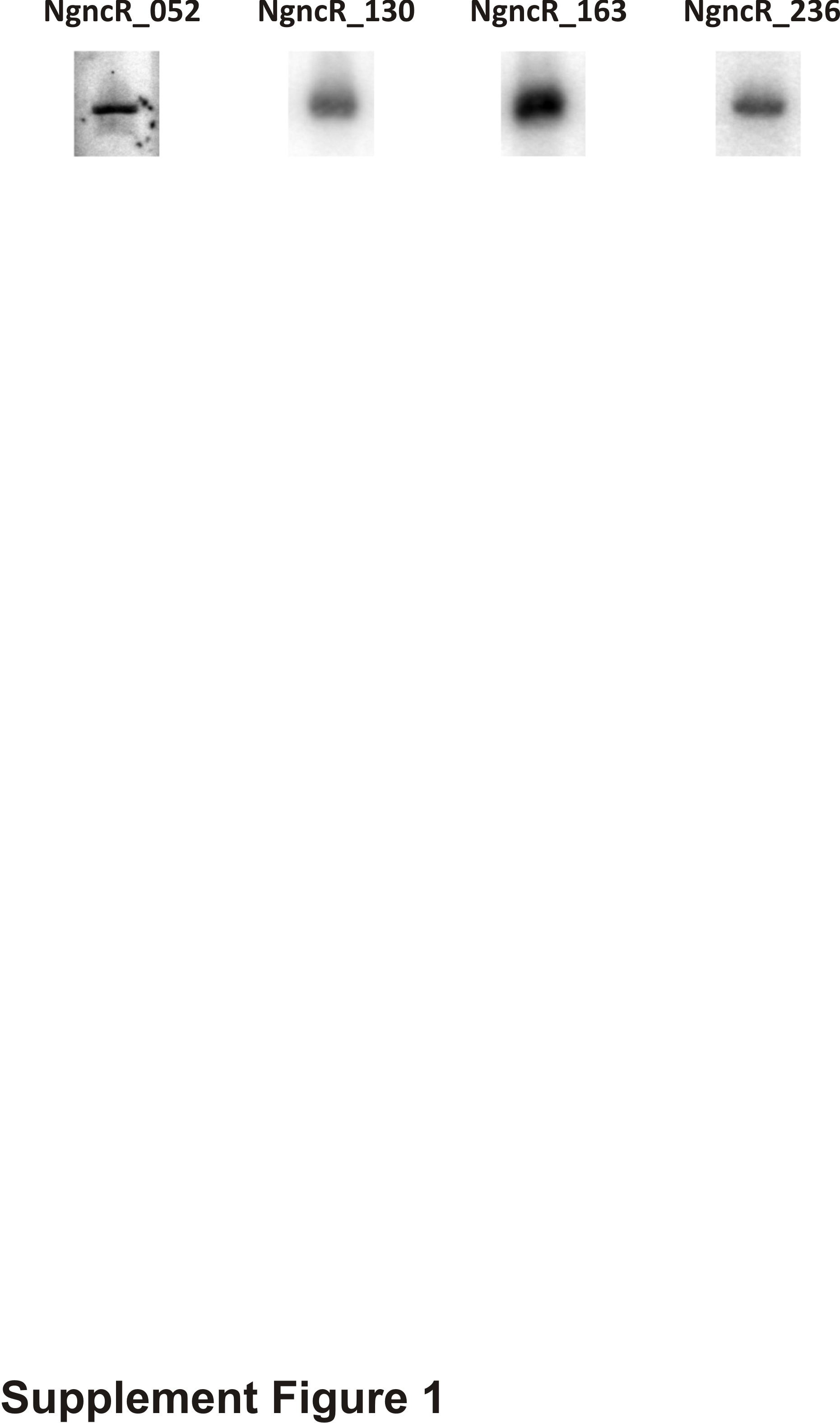

Supplement: SUPPLEMENTARY DATA [file supp_gku762_nar-01255-z-2014-File007.zip › NAR-01255-Z-2014.R1 Suppl files/Figure_S1.tif]

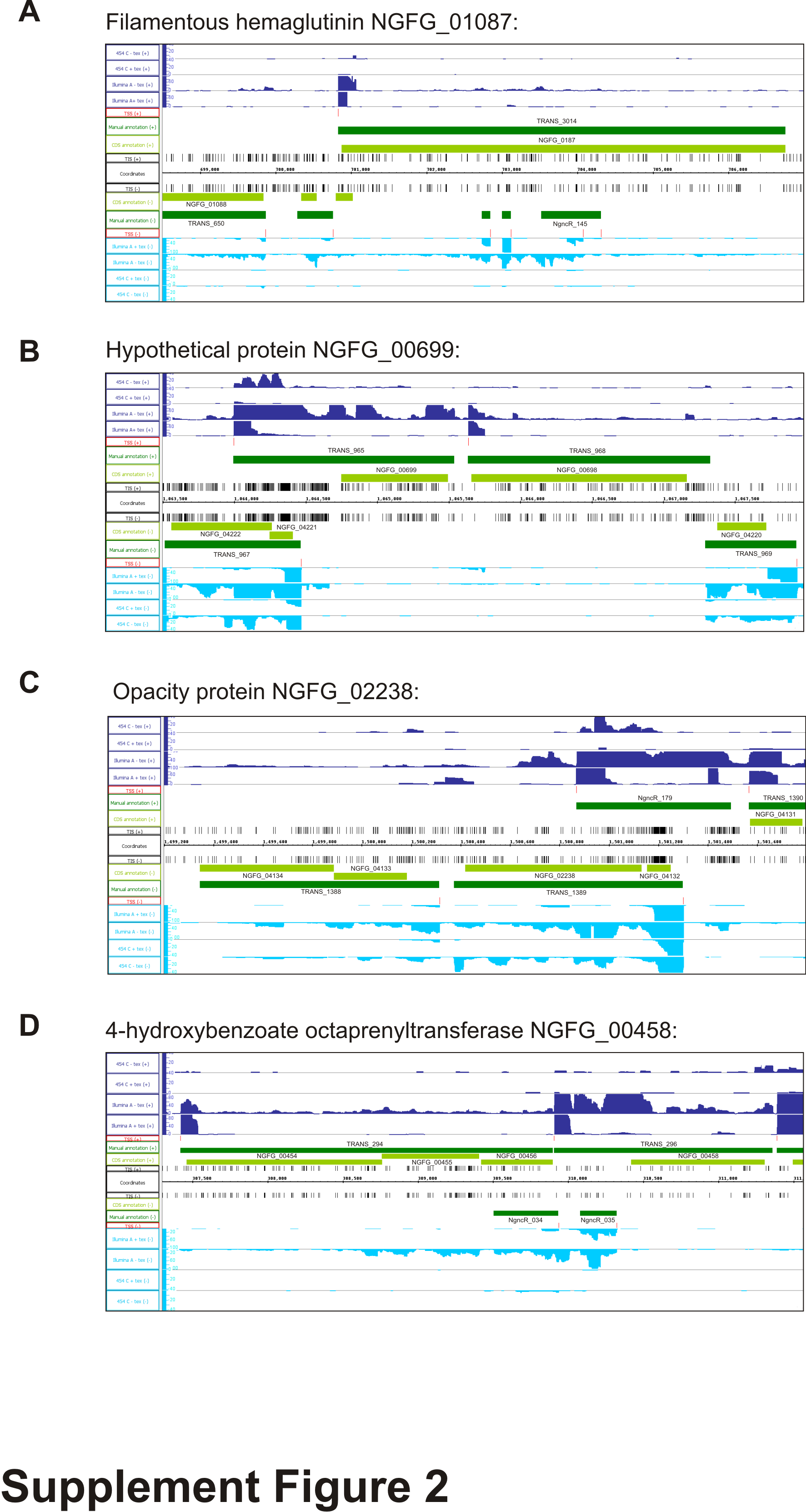

Supplement: SUPPLEMENTARY DATA [file supp_gku762_nar-01255-z-2014-File007.zip › NAR-01255-Z-2014.R1 Suppl files/Figure_S2.tif]

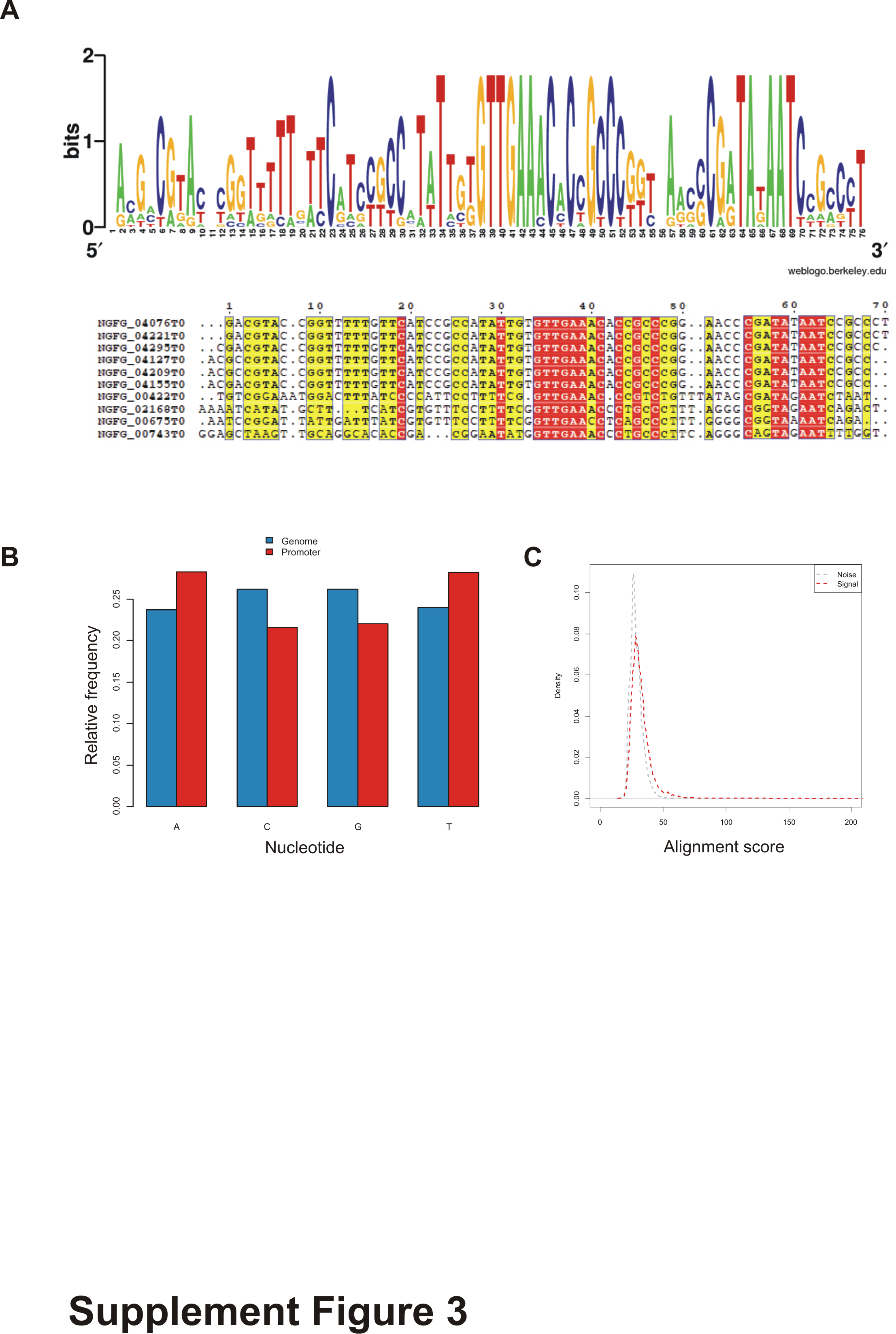

Supplement: SUPPLEMENTARY DATA [file supp_gku762_nar-01255-z-2014-File007.zip › NAR-01255-Z-2014.R1 Suppl files/Figure_S3.tif]

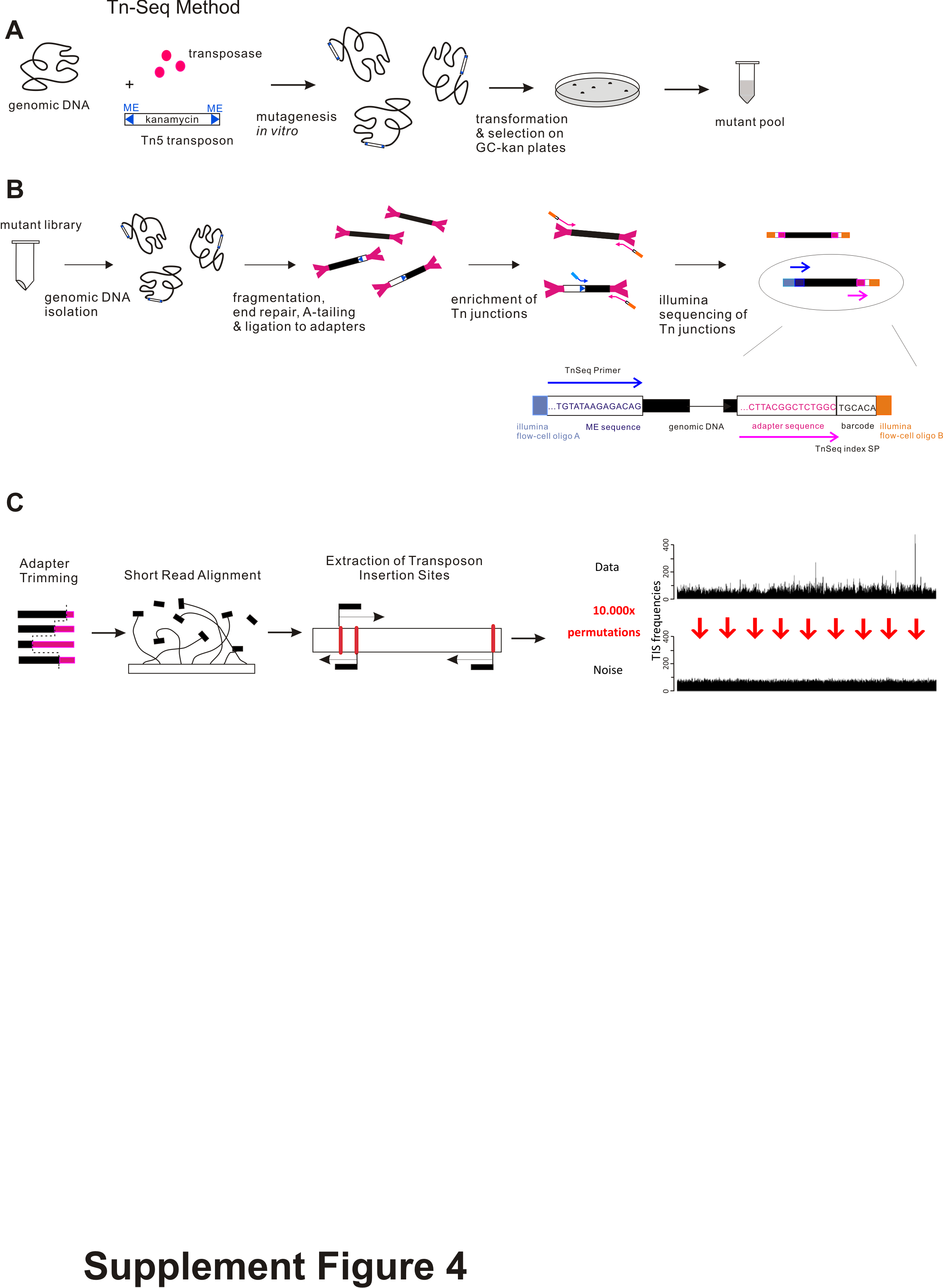

Supplement: SUPPLEMENTARY DATA [file supp_gku762_nar-01255-z-2014-File007.zip › NAR-01255-Z-2014.R1 Suppl files/Figure_S4.tif]

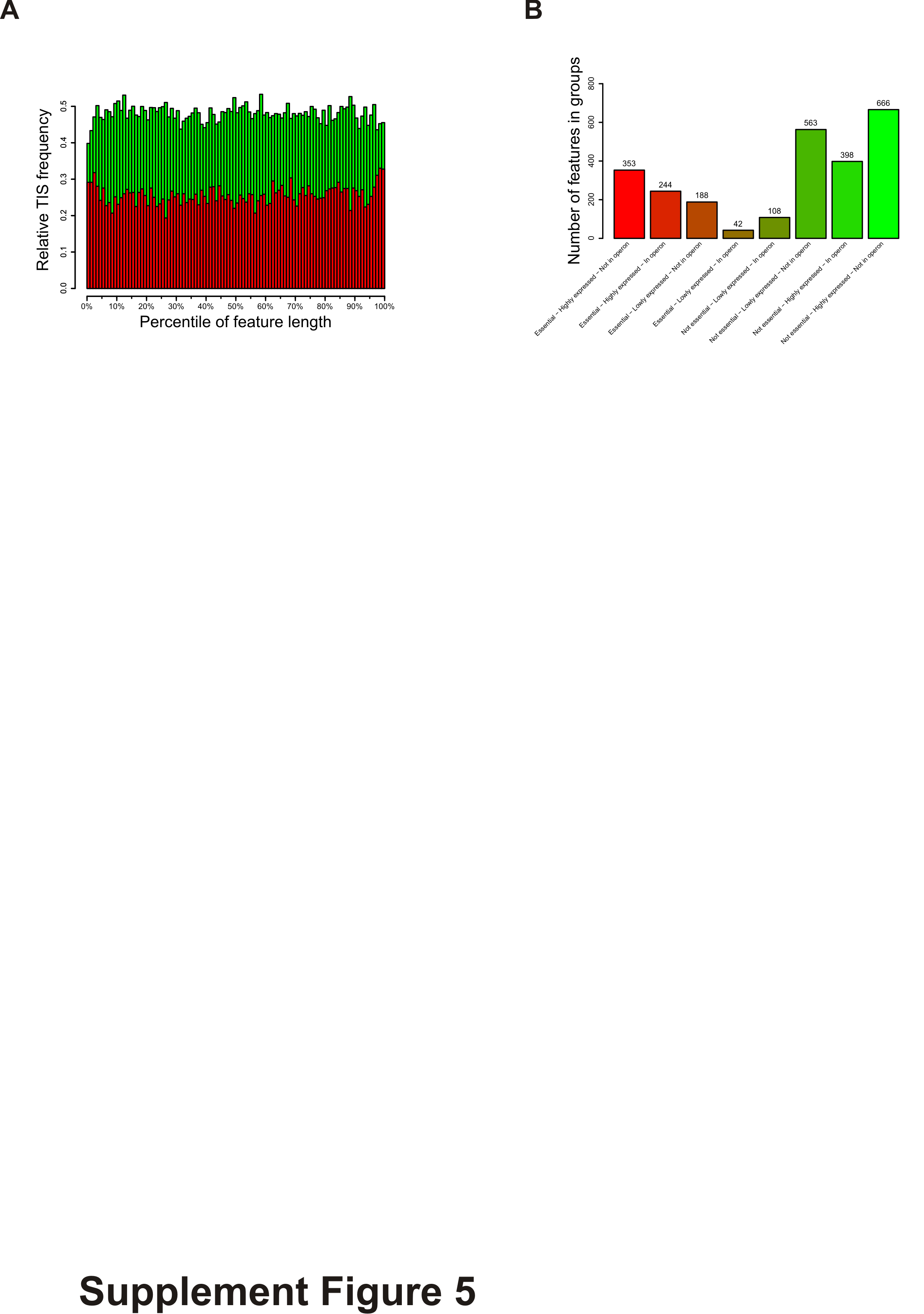

Supplement: SUPPLEMENTARY DATA [file supp_gku762_nar-01255-z-2014-File007.zip › NAR-01255-Z-2014.R1 Suppl files/Figure_S5.tif]

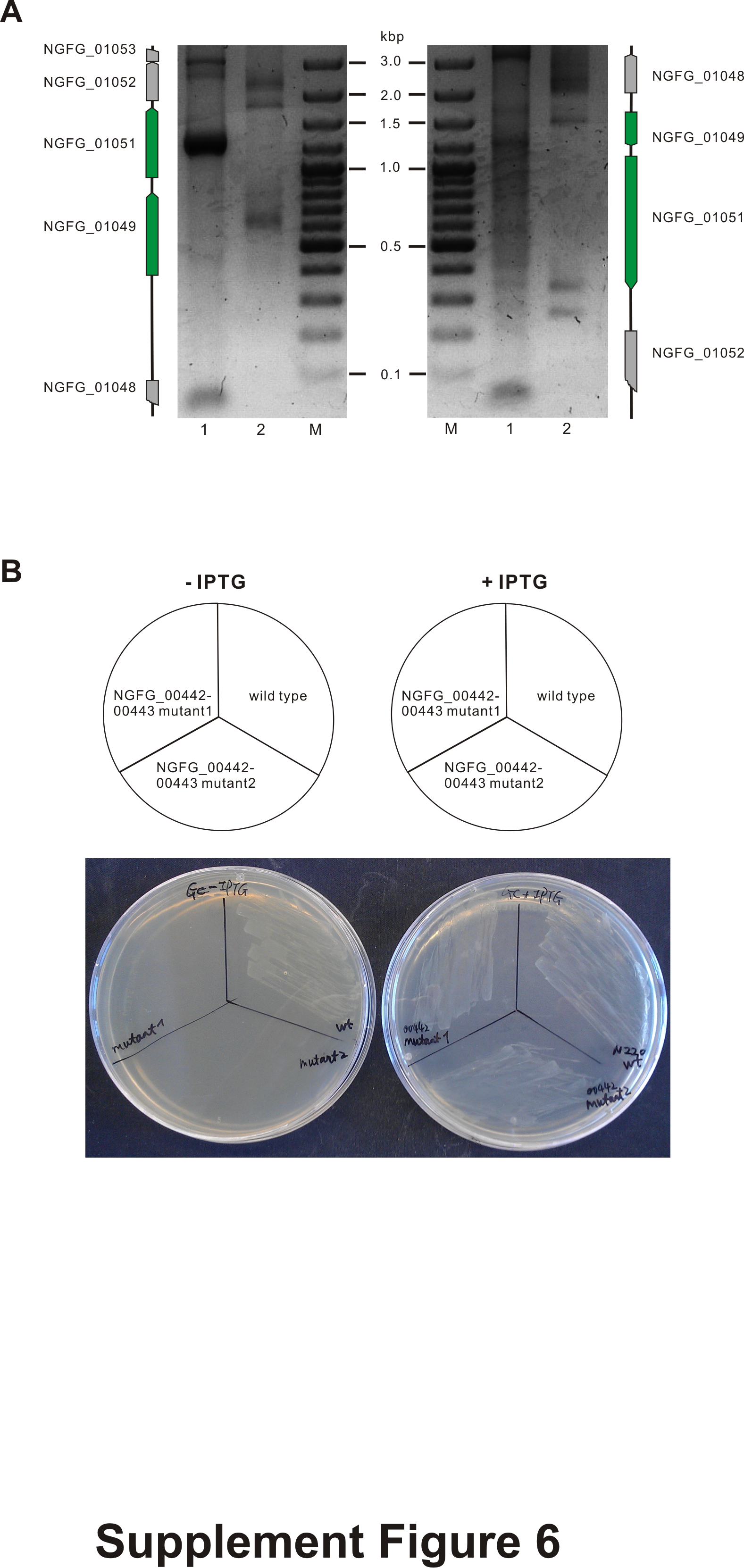

Supplement: SUPPLEMENTARY DATA [file supp_gku762_nar-01255-z-2014-File007.zip › NAR-01255-Z-2014.R1 Suppl files/Figure_S6.tif]
